# Social-emotional well-being in the context of health care: concept analysis^1^

**DOI:** 10.1590/1518-8345.8071.4798

**Published:** 2026-06-15

**Authors:** Cristiano de Oliveira Ribeiro, Luciana Puchalski Kalinke, Nuno Filipe de Oliveira Gil Salgado, Luciana de Alcantara Nogueira, Daniela Lourenço Pinto, Tereza Maria Mendes Diniz de Andrade Barroso

**Affiliations:** 1 Universidade Federal do Paraná, Campus Jardim Botânico, Curitiba, PR, Brazil.; 2 Scholarship holder at the Coordenação de Aperfeiçoamento de Pessoal de Nível Superior (CAPES), Brazil.; 3 Escola Superior de Enfermagem de Coimbra, Unidade de Investigação, Coimbra, CO, Portugal.

**Keywords:** Mental Health, Quality of Life, Delivery of Health Care, Concept Formation, Personal Satisfaction, Patient-Centered Care.

## Abstract

**Objective:**

to analyze the concept of socio-emotional well-being in the context of health care

**Method:**

concept analysis theoretical study based on the method of Walker and Avant, organized into eight stages, through scoping review. Seven data sources were searched, resulting in the selection of seven studies for the literature corpus.

**Results:**

social-emotional well-being is characterized by essential attributes such as emotional balance, perceived social support, feeling of control over one’s life, and emotional regulation skills. Antecedent factors included resilience, social support, self-knowledge, supportive environment, emotional stability, autonomy and decision-making and positive interpersonal bonds. Consequent factors included improved quality of life, increased social engagement, reduced psychosocial symptoms, and better performance in daily activities.

**Conclusion:**

the study showed that social-emotional well-being is a multifaceted concept that transcends individual mental health, encompassing social, emotional and cultural dimensions. Integrating social-emotional well-being into health care requires interdisciplinary approaches that consider individual and social factors, promoting holistic and patient-centered health care.

## Highlights

**(1)** Social-emotional well-being is multifaceted and multidimensional. **(2)** Social-emotional well-being contributes toward the improvement of quality of life. **(3)** Having emotional balance is key to dealing with emotions adaptively. **(4)** Social support contributes directly to emotional resilience. **(5)** Measuring social-emotional well-being involves a conceptual and methodological challenge

## Introduction

Well-being has been given increasing relevance in the health care field, especially as consideration as to care extends beyond purely biological aspects and begins to encompass emotional and social dimensions^1^. This holistic perspective favors more humane and person-centered practices, with a direct impact on the quality of the care provided.

Understanding well-being implies recognizing that mental health is not restricted to the absence of disorders, but involves emotional balance, psychological well-being and the ability to face the challenges of everyday life. Thus, it is intrinsically associated with each individual’s ability to cope with stress, work productively and contribute to their community^2^ ^-^ ^3^.

The World Health Organization (WHO)^4^ defines mental health as “a state of mental well-being that enables people to cope with the stresses of life, realize their abilities, learn and work well, and contribute to their community.” This concept encompasses emotional, psychological and social dimensions, directly influencing how people think, feel, act, cope with stress, build interpersonal relationships and make healthy decisions, which are essential and inseparable components of well-being^3^.

Over time, the concept of well-being has evolved from a view restricted to the absence of disease to more complex approaches, such as subjective well-being and psychological well-being, which consider, respectively, personal satisfaction and the full actualization of human potential^3^. This evolution shows that both the experience of positive emotions and the pursuit of purpose and growth are essential for a fulfilling life. However, the complexity of well-being goes beyond these individual dimensions, also incorporating social and relational aspects^5^.

Within this broader context, the concept of social-emotional well-being has arisen as an integrative perspective, emphasizing the interaction between social and emotional factors. This construct encompasses the ability to cope with daily challenges, develop resilience and maintain healthy relationships^6^. This perspective reflects a deeper and more multifaceted understanding of this phenomenon, conceiving social-emotional well-being as a balance between emotional regulation, the establishment of social relationships and a sense of purpose in life^7^. These approaches reinforce the understanding of social-emotional well-being as a multidimensional construct, essential to the integral development and quality of life (QoL) of individuals.

Despite the increasing use of the concept of social-emotional well-being in the literature, there is still no consensus on its definition, which limits its theoretical and practical application. The complexity of this construct requires researchers and health care professionals to consider subjective experiences, cultural aspects, social influences and family dynamics when developing health care promotion and prevention strategies.

In addition, the variety of terminologies used in the literature shows the need for a uniform definition, fundamental to guide both clinical practice and research. In this context, it is essential to clarify the concept of social-emotional well-being, which underlies the objective of this study: to analyze the concept of social-emotional well-being in the context of health care. 

## Method

### Study type

Concept analysis theoretical study based on the model proposed by Walker and Avant^8^ through scoping review^9^. Concept analysis is a systematic approach widely used in nursing research to clarify complex concepts and broaden their application in clinical practice^10^.

The model^8^ comprises eight stages: 1) concept selection, which consists of choosing the phenomenon to be studied; 2) determination of objectives, when the purposes of the analysis are defined; 3) identification of possible uses, which explores how the concept is employed in the literature; 4) definition of essential attributes, which establishes the main characteristics of the concept; 5) identification of a model case, which exemplifies the concept in a clear and representative manner; 6) analysis of other cases, including borderline, related, and contrary cases, to show differences and similarities; 7) identification of antecedent and consequent factors, which precede and result from the concept; and 8) definition of empirical benchmarks, which are indicators used to measure the concept in practice.

To support this analysis, we incorporated a scoping review^9^ (stage 3), registered in the Open Science Framework (https://osf.io/h5zr6/) under DOI 10.17605/OSF.IO/H5ZR6, which supported the subsequent stages of the model.

### Data collection

For formulation of the research question, we used the PCC mnemonic: Population - Unspecified; Concept - Social-emotional well-being; Context - Health care. Based on these definitions, the following guiding question was established: How is the concept of social-emotional well-being approached in the context of health care? The searches were performed in November and December 2024, with the primary strategy developed in conjunction with a professional librarian and adjusted for the eight data sources [PubMed, EMBASE (Excerpta Medica dataBASE), Web of Science, Cumulative Index to Nursing and Allied Health Literature (CINAHL), MEDLINE (Medical Literature Analysis and Retrieval System Online), Scopus (Elsevier), Virtual Health Library (VHL) and Google^®^ Scholar, combined with Boolean operators AND and OR (Figure 1).

**Figure 1:** Search strategies. Curitiba, PR, Brazil, 2025

**Data source**	**Search strategy**
VHL*	(“Bem-estar socioemocional” OR “Bienestar socioemocional” OR “Socioemotional well-being” OR “Bem-estar” OR “Bienestar” OR “Well-being”) AND (“Cuidados de saúde” OR “Cuidados de salud” OR “Atenção à saúde” OR “Atención a la salud”)
EMBASE^†^	(‘Socioemotional well-being’:ti,ab,kw OR ‘Well-being’:ti,ab,kw)) AND (‘Health care’:ti,ab,kw OR ‘Health care’/exp OR ‘Healthcare’:ti,ab,kw OR ‘Health services’:ti,ab,kw OR ‘Health services’/exp OR ‘Delivery of health care’:ti,ab,kw OR ‘Delivery of health care’/exp)
MEDLINE^‡^	(‘Socioemotional well-being’:ti,ab,kw OR ‘Well-being’:ti,ab,kw)) AND (‘Health care’:ti,ab,kw OR ‘Health care’/exp OR ‘Healthcare’:ti,ab,kw OR ‘Health services’:ti,ab,kw OR ‘Health services’/exp OR ‘Delivery of health care’:ti,ab,kw OR ‘Delivery of health care’/exp)
PubMed	(“Socioemotional well-being”[Title/Abstract] OR “Well-being”[Title/Abstract]) AND (“Health care”[MeSH Terms] OR “Health care”[Title/Abstract] OR “Healthcare”[Title/Abstract] OR “Health services”[MeSH Terms] OR “Health services”[Title/Abstract] OR “Delivery of health care”[MeSH Terms] OR “Delivery of health care”[Title/Abstract])
Scopus (Elsevier)	TITLE-ABS-KEY((“Socioemotional well-being” OR “Well-being”) AND TITLE-ABS-KEY (“Health care” OR “Healthcare” OR “Health services” OR “Delivery of health care”))
Web of Science	ALL=(“Socioemotional well-being” OR “Well-being”) AND ALL=(“Health care” OR “Healthcare” OR “Health services” OR “Delivery of health care”)
CINAHL^§^	(“Bem-estar socioemocional” OR “Bienestar socioemocional” OR “Socioemotional well-being” OR “Bem-estar” OR “Bienestar” OR “Well-being”) AND (“Cuidados de saúde” OR “Cuidados de salud” OR “Atenção à saúde” OR “Atención a la salud”)
Google Scholar	(“Bem-estar socioemocional” OR “Bienestar socioemocional” OR “Socioemotional well-being” OR “Bem-estar” OR “Bienestar” OR “Well-being”) AND (“Cuidados de saúde” OR “Cuidados de salud” OR “Atenção à saúde” OR “Atención a la salud”)

*VHL = Virtual Health Library; ^†^EMBASE = Excerpta Medica database; ^‡^MEDLINE = Medical Literature Analysis and Retrieval System Online; ^§^CINAHL = Cumulative Index to Nursing and Allied Health Literature

### Selection criteria

The delimited inclusion criteria were: studies accessible in full, published between 2014 and 2024, a period defined to cover recent productions, in Portuguese, Spanish and English; which contained the expression “social-emotional well-being” in the title, abstract or keywords, to ensure the relevance of the articles to the central theme of the research. The following studies were excluded: letters to the editor, editorials, abstracts and opinion studies and duplicates.

All identified articles were exported to the Rayyan^®^ review manager. Two reviewers blindly evaluated titles, abstracts, and descriptors based on the eligibility criteria. Disagreements were resolved by a third reviewer. In addition to selection in the databases, a manual search was performed in the reference lists of the included studies. 

### Study selection, data processing and analysis

The search found 13,728 studies, of which 1,883 were duplicates. After screening titles, abstracts and keywords related to the term social-emotional well-being, 11,785 were excluded. There were 60 studies left for full reading, of which 53 were discarded for not meeting the inclusion criteria. Only articles that explicitly addressed the concept of social-emotional well-being were considered, resulting in seven studies included in the final review.

After selection, the studies were organized as follows: author and year, country, population and sample, main objective, concept of social-emotional well-being in the context of health care, and observed dimensions, as illustrated in Figure 2. The essential social-emotional well-being attributes were defined through the following question: *What are the fundamental characteristics that define social-emotional well-being?* To identify its antecedent factors, the following question was asked: *What events precede social-emotional well-being?* Finally, to determine its consequent factors, the following question was considered: *What events result from social-emotional well-being?*

**Figure 2:** Characterization of selected studies. Curitiba, PR, Brazil, 2025

Author and year	Country	Population and sample	Main objective	Concept of social-emotional well-being in the context of health care	Observed dimensions
Ashton, et al., 2024^11^.	New Zealand	Parents (n=257) and children (n=285).	Assess the program’s long-term impact (12 months) when implemented in the community by trained facilitators.	Positive effect of community physical activities on social-emotional well-being, family bonds and mental health.	Strengthening family bonds; reducing stress; promoting emotional support; creating a positive and community environment.
Tunalilar, et al., 2024^12^.	USA*	Adult foster care providers (2,027).	Monitor family engagement changes among Oregon Adult Foster Home residents following the COVID-19 pandemic.	Effect of reduced family engagement on the residents’ social-emotional well-being.	Dependence on family engagement; feelings of loneliness; stress in the face of lacking support; importance of continuity of affective bonds.
Us, et al., 2024^1^.	Turkey	Children (n=600) of health care providers.	Explore social-emotional and behavioral issues of children of mothers that are health care providers.	Emotional impact due to the difficulties experienced by the children, associated with family and social stress.	Child vulnerability; impact of family and social stress; direct influence of maternal work on children’s emotional health.
Timmer, et al., 2022^13^.	Australia, Switzerland, USA* and UK^†^	Review	Provide evidence-based recommendations to ensure inclusion of social-emotional well-being in audiologic rehabilitation clinical practice.	Focus on the impact of hearing loss on social-emotional well-being, including self-esteem, social exclusion, and mental health.	Reduced self-esteem; social exclusion; anxiety and depression associated with hearing loss; relevance of emotional support in clinical care.
Siette, et al., 2021^14^.	Australia	Older adults (n=21) provided home care services.	To investigate the impact of the COVID-19 pandemic on the quality of life and social networks of older adults served in community care services.	Social-emotional well-being affected by social isolation and support networks.	Social isolation; loneliness; depression; importance of support networks to maintain well-being.
Fenwick, et al., 2020^15^.	Singapore and Australia	Adults (n=220) with diabetic retinopathy.	Validate a Diabetic Retinopathy Utility Index.	Assessment of how vision loss affects social-emotional well-being, self-esteem, social relationships and quality of life.	Self-esteem; social relationships; perceived loss and limitations; impact of chronic disease on daily life.
Pruner, et al., 2020^16^.	USA*	Adoptive/biological parents (n=25) of children with or at risk of FASD^‡^.	Identify characteristics of the early intervention practice that are both favorable and challenging for parents.	Parents’ social-emotional well-being; influence of early interventions on reducing stress and anxiety.	Parents’ emotional balance; influence of family dynamics; need for support and strategies for coping; relation between emotional health and care.

*USA = United States of America, †UK = United Kingdom; ‡FASD = Fetal Alcohol Spectrum Disorders

### Use of artificial intelligence 

**Figure 5:**
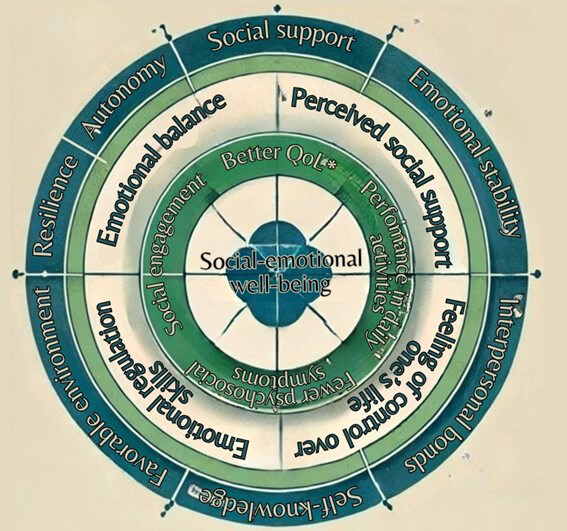
Conceptual diagram of social-emotional well-being. Curitiba, PR, Brazil, 2025

Source: OpenAI *QoL = Quality of life

The authors declare the use of ChatGPT (https://chatgpt.com) for preparation of Figure 5, which presents a conceptual diagram illustrating the relations between factors associated with social-emotional well-being. The data and figure were edited by the authors for improvement and enhancement.

## Results

The results are presented following the concept analysis stages according to Walker and Avant^8^.

### Selection and objective

“Well-being” is often defined as a state of physical, mental and social balance, in which the individual feels healthy, comfortable and in harmony with himself and the environment. This state involves emotional and psychological aspects, such as happiness, security and fulfillment, as well as physical components, such as health and vitality^5^.

Additionally, well-being can extend to the social context, encompassing healthy interpersonal relationships and a sense of belonging to the community. In general, it represents the condition of being well holistically, including the absence of suffering, a good QoL and the ability to face challenges adaptively and positively^5^.

Although the WHO provides no specific definition for “social-emotional well-being,” the concept appears to be closely related to mental health, as it involves the ability to deal with emotions, establish healthy interpersonal relationships, and maintain emotional balance. According to the Australian Institute of Health and Welfare^17^, social-emotional well-being is described as an essential part of mental and social well-being, which goes beyond the absence of disease and includes the ability to maintain positive relationships, manage emotions and participate actively in the community, encompassing emotional resilience, a sense of belonging, and social support.

The lack of consensus in the definitions of social-emotional well-being shows its complexity and reinforces the need for a clear and contextualized conceptualization. This lack of consensus limits its application in research and interventions, hindering comparisons and generalizations. Establishing a definition that is consistent, flexible, and applicable to different contexts is essential to consolidate the concept, guide effective caregiving practices, and promote social-emotional well-being broadly and meaningfully.

### Identification of possible uses of the concept

This section identifies the possible uses of the concept of well-being, based on the seven studies selected in stage three, which explore its applications, characteristics and practical implications in different contexts.

A study^1^ conducted in Turkey used the concept of social-emotional well-being to investigate how stress and work overload experienced by health care mothers during the COVID-19 pandemic affected the emotional and behavioral balance of their children. The study noted that the children’s social-emotional well-being was compromised, resulting in higher rates of anxiety, depression, and behavioral issues. Thus, the concept of social-emotional well-being was applied to understand how external factors, such as parental stress, impact children’s mental health and reinforce the need for policies to support families in crisis situations.

In a different context, a study^15^ in Singapore and Australia indirectly addressed social-emotional well-being by validating a utility index for patients with diabetic retinopathy. Although the primary focus was on health-related QoL, the study showed that vision loss can lead to social isolation and depression, impacting the patients’ social-emotional well-being. In this case, the concept was used to emphasize the importance of considering emotional and social aspects in the treatment of chronic conditions.

In the United States of America (USA), a study^16^ explored the experiences of parents of children with fetal alcohol spectrum disorder (FASD), using the concept of social-emotional well-being to understand how stress and emotional overload affect the mental health of parents. The study showed that several parents face feelings of guilt, stress, and isolation, compromising their emotional balance. Here, the concept was applied to emphasize the need for psychological and practical support for parents, promoting a healthier family environment.

In Australia, a study^14^ addressed the impact of the COVID-19 pandemic on the QoL of older adults receiving community care, using the concept of social-emotional well-being to explore how social isolation and interruption of services affected the mental health of older adults. The study showed that many older adults reported loneliness and depression, underscoring the importance of strategies to maintain social contact and emotional support. In this context, the concept was applied to emphasize the need for integrated approaches that consider both physical and emotional care.

Another study^13^, a collaboration between Australia, Switzerland, the USA and the UK, focused on the social-emotional well-being of adults with hearing loss. The concept was used to discuss how hearing loss can lead to social isolation and depression, thus impacting QoL. The study recommended the introduction of social-emotional well-being promotion strategies, such as psychological counseling and social support, in audiological rehabilitation programs. The concept was applied to show the importance of a holistic approach in the treatment of chronic conditions.

In the USA, a study^12^ analyzed family engagement among residents of adult foster care homes, using the concept of social-emotional well-being to explore how the reduced family contact during the pandemic affected the mental health of the residents. The study showed that several residents reported feelings of abandonment and loneliness, showing the importance of family engagement for emotional balance. In this case, the concept was applied to reinforce the need for policies that strengthen family bonds and promote social-emotional well-being.

In New Zealand, a study^11^ assessed the results of a community-based physical activity program among parents and daughters, using the concept of social-emotional well-being to explore how social interaction and physical activity impact the mental and emotional health of the participants. The study showed that the program promoted significant improvements in social-emotional well-being, strengthening family bonds and reducing stress. The concept was applied to emphasize the importance of programs that integrate physical activity and social interaction to promote mental health.

By analyzing these studies, it is reinforced that social-emotional well-being is a multidimensional concept, essential for QoL in different phases and contexts. From the impact of the pandemic on child mental health to the challenges faced by older adults and adults with chronic conditions, emotional and social balance arises as a crucial factor for human development.

### Essential attributes

Defining essential attributes is a fundamental step in concept analysis, as these characteristics clarify the essence of the concept, enabling its recognition, differentiation and validation of its applicability in different contexts^8^. Analysis of the selected studies showed the essential attributes of social-emotional well-being.

Emotional balance

Emotional balance refers to the ability to deal with emotions in a stable and adaptive manner, minimizing negative impacts of anxiety, stress, and sadness. It is essential for maintaining mental health and directly influences how individuals cope with everyday challenges^14^ ^,^ ^16^.

The relevance of emotional balance is evident in different contexts. A study^14^ that followed older adults in community care during the pandemic used mental health scales and semi-structured interviews to analyze the relation between emotional balance and QoL. The results indicated that older adults with higher emotional balance had lower levels of loneliness and depression, demonstrating the relevance of this attribute for social-emotional well-being, especially in situations of vulnerability.

Beyond the individual impact, emotional balance also influences the family environment. A study^16^ on parents of children with or at risk of FASD found that the emotional balance of parents directly impacts the well-being of families. Parents with higher emotional control showed better adaptation to the difficulties faced, contributing to the emotional health of all family members. These findings reinforce that emotional balance strengthens the individual and plays a crucial role in interpersonal relationships and family dynamics. 

*Perceived social support*

Perceived social support is related to the feeling of support received from family members, friends and communities. It plays a central role in emotional resilience and coping with difficulties, fostering a sense of belonging and security. In addition, feeling supported is directly associated with confidence and emotional stability^11^ ^,^ ^13^.

Perceived social support is a determining factor for emotional well-being, especially in vulnerable groups. In patients with hearing loss, feeling supported reduced stress levels and facilitated adaptation to limitations, as demonstrated by a study using questionnaires and psychological tests^13^. In the family context, support was essential for the social-emotional well-being of foster care home residents, while its absence, especially during the COVID-19 pandemic, was associated with higher stress and emotional difficulties^12^.

Beyond the family environment, community support also plays a key role. A study that analyzed the participation of parents and children in a community physical activity program demonstrated that social support strengthened family bonds and promoted the feeling of belonging, thereby contributing to the participants’ social-emotional well-being^11^.

*Feeling of control over one’s life*

The feeling of control over one’s life is related to the perceived autonomy and ability to make decisions that influence one’s well-being. This attribute is strongly associated with self-confidence, independence and personal satisfaction^15^.

The perceived control over one’s life has shown a significant impact on adaptation to adverse conditions. A study^15^ with patients with diabetic retinopathy, conducted through discrete choice experiments and QoL reports, observed that those who felt in command of their own health-related decision-making reported higher satisfaction and a more positive adaptation to the chronic condition. This shows the importance of feeling in control for emotional well-being and resilience in the face of complicated health conditions.

In a similar context, a study^14^ with older adults under community care during the COVID-19 pandemic showed the relevance of autonomy in emotional health. Those who maintained a feeling of control over their lives had less anxiety and better QoL, suggesting that the ability to manage one’s life, especially in times of major uncertainty, is critical to maintaining emotional health.

*Emotional regulation skills*

Emotional regulation skills involve the ability to recognize, understand and manage emotions adaptively, enabling individuals to face challenges without developing disproportionate stress or anxiety responses^13^.

The relevance of this attribute was shown in different populations. A study^16^ with parents of children with FASD, based on interviews and emotional regulation scales, indicated that those with higher command of these skills had better adaptation to complex challenges, favoring psychological balance and resilience.

In addition, a review^13^ on the impact of hearing loss on adult social-emotional well-being showed that emotional regulation plays an essential role in adapting to hearing loss. Patients who showed greater ability to manage their emotions faced the challenges of the condition in a more positive manner, showing that the development of these skills can mitigate the negative impacts of physical limitations on QoL. Figure 3 shows the essential attributes under consideration. 

**Figure 3:** Essential attributes of social-emotional well-being. Curitiba, PR, Brazil, 2025

Attributes	Description	Related studies
Emotional balance	Emotional regulation skills.	Siette, et al. (2021): Older adults who maintained emotional balance reported less loneliness and depression^14)^.
Perceived social support	Sense of belonging and connection.	Timmer, et al. (2022): Patients with hearing loss who perceived social support had better emotional well-being^13^.
Feeling of control over one’s life	Autonomy and decision-making skills.	Fenwick, et al. (2020): Patients who felt control over their condition reported higher quality of life^15^.
Emotional regulation skills	Skills to identify and regulate emotions.	Pruner, et al. (2020): Parents who developed emotional regulation skills coped better with stress^16^.

These attributes are fundamental to social-emotional well-being and are manifested in different contexts. Perceived social support strengthens the sense of belonging and connection, while the feeling of control over one’s life promotes autonomy and reduces anxiety. Emotional balance enables one to deal with stressors adaptively, positively impacting mental health. In addition, emotion regulation skills enable individuals to manage their emotions effectively, preventing disproportionate reactions and promoting overall well-being.

### Model case

Cases are examples used to illustrate and clarify the concept under study. They help differentiate the concept from related terms and show how it can be applied in different contexts^8^.

The model case represents the complete manifestation of the concept of social-emotional well-being. L.P.M., 45 years old, a university professor recently diagnosed with early-stage breast cancer, has since the diagnosis had anxiety attacks that intensify at night. She initiated professional follow-up with psychotherapy, regular practice of guided meditation and participation in a community arts group in the community. Over the course of eight months, she developed essential attributes of social-emotional well-being. L.P.M. exhibits the ability to deal with complex situations associated with treatment, maintains significant bonds with family members and colleagues, perceives herself supported in her social network and expresses feelings of hope for the future. In addition, she presents a healthy regulation of emotions in the face of stressing situations, manages to re-signify her experience of illness, and maintains a satisfactory level of engagement in daily activities. As a result, she presents reduced emotional distress, better performance in daily activities, and higher QoL. This case reflects the full manifestation of the concept of social-emotional well-being.

### Analysis of other cases

#### Borderline case

The borderline case contains some but not all of the essential attributes of the concept^8^. L.G.P., 42 years old, diagnosed with depression triggered by labor stressors, initiated follow-up with cognitive-behavioral therapy and drug treatment. As a complement, they began to perform regular physical activities and dedicate themself to leisure practices that afford them pleasure. Despite the initial improvement, with more positive coping and a feeling of greater control over their own life, they still present emotional instability (depressed mood, irritability, anxiety) and difficulties in emotional regulation, especially in the face of work-related demands. Although depressive symptoms have decreased, significant psychological fluctuations persist. Therefore, this is a borderline case, in which some social-emotional well-being attributes are present, but not in an integral way.

#### Contrary case

The contrary case exemplifies the “not the concept,” that is, the total or almost total absence of the essential attributes of the concept^8^. C.O.R., 38 years old, who has recently moved from the countryside to São Paulo-SP, presents anxiety and depression disorders. Although he consulted a psychiatrist, he neither followed medical recommendations nor sought additional psychological or therapeutic follow-up. He manifests recurrent episodes of irritability, depressed mood, anxiety and feeling of lost control. He lives isolated and without support networks, remaining much of the time secluded in his room, without engaging in social or leisure activities. In this context, anxiety and depression symptoms are aggravated by the lack of social support and adequate coping strategies. Thus, low QoL is observed, with the absence of the essential attributes of social-emotional well-being.

#### Related case

The related case illustrates a situation that is associated with the concept of social-emotional well-being^8^. G.T., 28 years old, a sixth-year medical student, presents with chronic stress, insomnia and emotional instability for much of the school period. They believe they have an anxious and depressive syndrome. However, after the end of the tests, there is a significant improvement in the situation, due to the reduction of stress and the resumption of physical activities, leisure practices and social interaction with friends. This case is related to the concept, but does not fully cover its essential attributes, as there is no search for professional evaluation and therapeutic follow-up, essential for the complete development of social-emotional well-being.

### Antecedent and consequent factors

Antecedent factors refer to the causes or circumstances that precede the promotion of social-emotional well-being^8^. The analysis of the studies showed that several factors contribute to the development of this concept.

#### Resilience

The ability to overcome adversity and adapt to challenging circumstances plays an essential role in emotional balance and maintenance of social-emotional well-being^15^. More resilient individuals tend to have greater control over their own lives and develop more effective strategies to cope with stressful situations, which favors their adaptation to different contexts and contributes toward improving QoL^15^.

#### Social support

Emotional and practical support offered by family members, friends or community networks has a direct impact on reducing stress and strengthening well-being. The presence of a support network is a contributing factor when facing challenges and promotes a feeling of belonging, thus favoring emotional stability^14^. In addition, environments that foster social interaction strengthen interpersonal bonds and create favorable conditions for healthy social-emotional development^13^.

#### Self-knowledge 

People with a higher degree of self-knowledge deal better with daily challenges, avoiding impulsive or disproportionate responses. This factor is directly associated with the development of resilience and the strengthening of emotional skills, contributing toward a better adaptation to different life circumstances^16^.

#### Favorable environment 

A context that provides emotional security, social support, and access to adequate resources directly influences social-emotional well-being. Supportive environments promote greater emotional stability and facilitate the creation of positive interpersonal relationships. The presence of spaces that encourage healthy interactions and provide continuous support helps strengthen emotional regulation and the ability to face challenges^16^.

#### Emotional stability

Maintaining a balanced emotional state in the face of adverse situations is an essential factor for social-emotional well-being. Emotionally stable individuals have greater control over their actions and reactions, which favors a more positive adaptation to different circumstances^15^. This stability is related to the ability to regulate emotions and face challenges rationally and resiliently.

#### Autonomy and decision-making

The perceived control over one’s own life is directly associated with social-emotional well-being. Having autonomy to make decisions, especially in managing one’s own health and organizing daily life, reduces anxiety levels and promotes greater personal satisfaction. Feeling in command of one’s own choices boosts self-confidence and provides a greater feeling of actualization and purpose^15^.

#### Positive interpersonal bonds

Healthy relationships and meaningful social interactions are fundamental for social-emotional well-being. Interpersonal bonds based on trust and mutual support afford emotional security and contribute toward building a sense of belonging^14^. Environments that foster these connections strengthen the perception of social support and promote greater emotional stability over time.

The consequent factors of social-emotional well-being refer to the outcomes that arise as a consequence of this state^8^. 

#### Improved quality of life

Social-emotional well-being contributes to a more balanced general state, directly reflected in QoL. Individuals who show greater emotional stability and adequate social support face challenges with more resilience, exhibit greater personal satisfaction, and maintain a more positive outlook on life. This is noticeable in the adaptation to chronic health conditions, in coping with family difficulties and in the management of day-to-day stress^14^.

#### Increased social engagement

People who experience a balanced emotional state tend to seek and maintain social connections more actively, participating in community activities, establishing stronger bonds with family members and friends, and developing a sense of belonging. This interaction favors the exchange of support and creates a virtuous cycle in which social contact reinforces emotional well-being^11^.

#### Reduced psychosocial symptoms

The ability to regulate emotions effectively and the presence of a reliable support network enables individuals to better cope with adverse situations while minimizing negative mental health impacts. In vulnerable groups, such as older adults, chronically ill individuals or those facing significant changes in their lives, the presence of social-emotional well-being can be a determining factor for the prevention of psychological disorders^13^.

#### Improved performance in daily activities

Individuals with higher emotional balance exhibit greater motivation, concentration and adaptability, which is reflected in better performance in the school, work and family interactions. Children and adults with high social-emotional well-being have greater engagement in their tasks and a more positive approach to challenges, favoring a more productive and efficient functioning^1^.

The consequent factors of social-emotional well-being show its broad benefits, encompassing emotional, social and functional aspects. Psychological stability favors active participation in social life and the maintenance of good QoL, creating a positive cycle in which emotionally balanced individuals tend to develop healthier relationships and face challenges more confidently. Thus, fostering and strengthening social-emotional well-being becomes an essential strategy to improve the mental health and general well-being of the population. 

Figure 4 shows the antecedent and consequent factors of social-emotional well-being.

**Figure 4:** Antecedent and consequent factors of social-emotional well-being. Curitiba, PR, Brazil, 2025

Category	Elements	Description	Related studies
Antecedent factors	Resilience	Dealing with adversity, such as parental stress in the pandemic.	Us, et al. (2024): Resilience mitigated stress effects in children of mothers who are health care providers^1^.
	Social support	Support networks promote emotional stability.	Tunalilar, et al. (2024): Family support essential for well-being in adult foster care homes^12^.
	Self-knowledge	Emotional awareness assists in stress regulation.	Pruner, et al. (2020): Parents of children with fetal alcohol spectrum disorder noted the importance of self-knowledge^16^.
	Favorable environment	Safe and resourceful contexts strengthen social-emotional well-being.	Ashton, et al. (2024): Community programs promote a positive environment^11^.
	Emotional stability	Ability to maintain balance in the face of emotions.	Fenwick, et al. (2020): Emotional regulation in adverse situations favors positive adaptation in different circumstances^15^.
	Autonomy	Control over decision-making increases confidence and purpose.	Fenwick, et al. (2020): Autonomy strengthens self-esteem and satisfaction^15^.
	Positive interpersonal bonds	Healthy and supportive relationships that contribute to social-emotional well-being.	Siette, et al. (2021): Older adults with significant social support and interactions have greater emotional stability over time^14^.
Consequent factors	Improved quality of life	Greater satisfaction and well-being.	Siette, et al. (2021): Older adults with support had better quality of life^14^.
	Social engagement	Increased social interaction and participation.	Ashton, et al. (2024): Activity programs fostered greater engagement^11^.
	Reduced psychological symptoms	Less anxiety and depression.	Timmer, et al. (2022): Patients with support reported emotional improvement^13^.
	Improved performance	Increased productivity and functionality.	Us, et al. (2024): Children with higher well-being had better school performance^1^_._

Figure 5 presents a conceptual diagram that illustrates the relations between factors associated with social-emotional well-being, organized into three levels: antecedent factors, attributes and consequent factors.

## Discussion

### Definition of empirical frameworks

Empirical frameworks are the measurement tools that demonstrate the occurrence of the concept, requiring the identification of observable indicators, measurement methods and manifestation patterns^8^. 

The analyzed studies provide evidence on how social-emotional well-being is manifested and influenced by external factors, providing a more structured understanding of its fundamental characteristics. However, its measurement still faces conceptual and methodological challenges, as there is no instrument that fully captures the complexity of the experiences associated with this concept.

Mental health and well-being are multidetermined constructs, informed by biological, social and cultural factors that influence the perception, emotional experience, and coping and social interaction strategies of individuals. As each cultural context defines its own parameters for what constitutes mental health, sensitivity to these nuances is imperative for the relevance and effectiveness of any intervention. This inherent complexity requires the development of assessment instruments capable of apprehending its multiple dimensions, enabling a comprehensive and properly contextualized measurement of the concept^2^.

The reviewed studies adopted standardized instruments to assess different dimensions of social-emotional well-being, including QoL, social support, and emotional well-being. The Lubben Social Network Scale (LSNS-6) was used to measure perceived social support, focusing on family and friend support networks. In turn, the EQ-5D-5L (EuroQoL-5D-5L) assessed health-related quality of life (HRQoL) in five dimensions: mobility, self-care, pain/discomfort, usual activities, and anxiety/depression symptoms^14^.

Other instruments were applied to measure specific aspects of social-emotional well-being in different populations. The Brief Infant-Toddler Social and Emotional Assessment (BITSEA) scale, for example, was used to assess the social and emotional health of children aged up to three years^1^. In the context of hearing aid, questionnaires such as the Patient Health Questionnaire-4 (PHQ-4), the Acceptance and Action Questionnaire-Adult Hearing Loss (AAQ-AHL), the Social Participation Restrictions Questionnaire (SPaRQ) and its reduced five-item version (SIM), in addition to the Hearing Handicap Inventory for the Elderly - Screening Version (HHIE-S), were recommended to investigate the impact of hearing loss on social-emotional well-being^13^.

In addition, a multicenter study^15^ incorporated social-emotional well-being into the development of the Disease-Related Utility Index (DRU-I), a scale aimed at assessing hearing-related QoL. In this scale, social-emotional well-being was considered one of the five central dimensions of patient QoL. However, the complexity of this construct requires a more in-depth approach, addressing its multiple dimensions and factors, providing a more comprehensive and accurate evaluation.

Importantly, none of the reviewed studies developed or applied a scale that objectively measures social-emotional well-being. The adopted tools assess correlated aspects, such as depression and anxiety symptoms and life satisfaction, but do not capture the construct comprehensively and specifically. This shows a gap in the literature regarding the direct measurement of social-emotional well-being.

The analysis of the reviewed studies shows that social-emotional well-being is a multidimensional construct, influenced by several factors, such as adaptation to stress^12^, quality of interpersonal relationships^15^ and social support^14^. In addition, concepts such as emotional resilience^16^, self-esteem^13^, family support^11^ and social adaptation^1^ are fundamental to promote social-emotional well-being, especially in adverse contexts, such as public health crises and chronic diseases. These factors have a significant impact on the mental health of individuals of different age groups and are essential for designing more effective and personalized interventions. 

Thus, integrating social-emotional well-being promotion strategies into health care practices and public policies is crucial, recognizing the complexity and interdependence of factors that support mental and emotional health throughout life^18^. The interventions should be adapted to the specific needs of each population, ensuring that emotional aspects are effectively incorporated into approaches geared toward health care and QoL.

A central point that emerges from the analysis is that, despite the different contexts in which it can be investigated - such as palliative care^19^, therapeutic diets^20^, chronic diseases^21^ or experiences of cultural minorities^22^ ^-^ ^24^ -, social-emotional well-being, in all cases, appears as a mediator of adaptation to illness and adversity. This reinforces the idea that health cannot be understood only in biomedical terms, but must also be understood as a phenomenon that integrates emotional processes, social relations and structural conditions of life.

Another relevant aspect is the relational dimension of social-emotional well-being. Studies in indigenous populations^22^ ^-^ ^24^ and clinical settings^19^ show that the quality of social connections, community support and cultural valorization play a central role in preserving social-emotional well-being. This perspective broadens the understanding of the construct, dissociating it from an individualized reading and associating it to an ecosystemic view, in which family, community and culture are determinants.

The discussion also indicates the importance of health equity as a determining factor of social-emotional well-being. Studies^21^ ^,^ ^25^ during the COVID-19 pandemic show how social determinants - such as income, access to services and social support - directly affect social-emotional well-being. These findings reinforce that public policies need to consider that social vulnerability translates not only into worse clinical outcomes, but also into greater emotional and social suffering, supporting the interdependence between structural inequality and mental health.

In this context, it is essential to recognize that the concept of well-being varies significantly between individuals and communities, informed by factors such as sociocultural context, personal experiences and collective values. Far from being a static or universal concept, well-being is configured as a dynamic and situated construction, whose understanding requires the integration of multiple perspectives. This semantic variability underlines the importance of approaches that take into consideration the particularities of each group^26^.

It should be noted that the analyzed studies show that social-emotional well-being should be understood as a multidimensional and dynamic construct, involving life experiences, cultural contexts and social conditions^3^. At the same time, these analyses indicate an important gap: the lack of specific instruments that directly measure this dimension. This methodological limitation does not invalidate the theoretical advances, but indicates the need for the development of more comprehensive measures that capture the complexity of the construct and provide inputs for health care practices and public policies geared toward promoting integral health^20^ ^,^ ^24^ ^-^ ^25^.

A limitation of this concept analysis lies in the lack of terminological and conceptual standardization in studies on social-emotional well-being. The employed definitions and terminologies show a diversity that hinders the comparison of results between different studies, compromising the construction of a cohesive conceptual foundation. Furthermore, the adopted time frame may have excluded relevant studies published outside the analyzed period, limiting the scope of the review. Similarly, the use of only Google^®^ Scholar as a source of gray literature may have limited access to complementary perspectives. 

## Conclusion

Social-emotional well-being is a multidimensional construct that goes beyond individual mental health, encompassing social, emotional and cultural interactions. Factors such as social support, interpersonal relationships, socio-cultural context and living conditions directly influence its manifestation and perception by individuals, showing the need for an integrated and contextualized understanding of the concept.

This concept analysis contributes toward consolidating the understanding of social-emotional well-being, supporting the design of health care public policies and interventions that integrate emotional, social and cultural aspects. Recognizing that the concept varies between different contexts and populations enables the promotion of more effective strategies geared toward integral health and QoL.

## Data Availability

All data generated or analysed during this study are included in this published article.
