# Supplementary material for: Social-emotional well-being in the context of health care: concept analysis
Source: Rev Lat Am Enfermagem. 2026 Jun 15;34:e4798. doi: 10.1590/1518-8345.8071.4798 (PMC13277358; doi:10.1590/1518-8345.8071.4798)
Supplement: Translation

# Translation: Bem-estar socioemocional no contexto do cuidado em saúde: análise de conceito^*^

**DOI:** 10.1590/1518-8345.8071.4799

**Authors:** Cristiano de Oliveira Ribeiro, Luciana Puchalski Kalinke, Nuno Filipe de Oliveira Gil Salgado, Luciana de Alcantara Nogueira, Daniela Lourenço Pinto, Tereza Maria Mendes Diniz de Andrade Barroso

**Affiliations:** 1 Universidade Federal do Paraná, Campus Jardim Botânico, Curitiba, PR, Brasil.; 2 Scholarship holder at the Coordenação de Aperfeiçoamento de Pessoal de Nível Superior (CAPES), Brasil.; 3 Escola Superior de Enfermagem de Coimbra, Unidade de Investigação, Coimbra, CO, Portugal.

**Keywords:** Saúde Mental, Qualidade de Vida, Atenção à Saúde, Formação de Conceito, Satisfação Pessoal, Assistência Centrada no Paciente

## Abstract

**Objetivo:**

analisar o conceito do bem-estar socioemocional no contexto do cuidado em saúde.

**Método:**

estudo teórico de análise de conceito orientado pelo método de Walker e Avant, organizado em oito etapas e operacionalizado mediante revisão de escopo. A busca se deu em sete fontes de dados, resultando na seleção de sete estudos para compor o corpus literário.

**Resultados:**

o bem-estar socioemocional é caracterizado por atributos essenciais como equilíbrio emocional, percepção de suporte social, sensação de controle sobre a própria vida e habilidades de regulação emocional. Os antecedentes incluíram resiliência, suporte social, autoconhecimento, ambiente favorável, estabilidade emocional, autonomia e tomada de decisão, além de vínculos interpessoais positivos. Como consequentes, observaram-se melhora na qualidade de vida, aumento do engajamento social, redução de sintomas psicossociais e melhor desempenho em atividades cotidianas.

**Conclusão:**

o estudo mostrou que o bem-estar socioemocional é um conceito multifacetado que transcende a saúde mental individual, abrangendo dimensões sociais, emocionais e culturais. Sua integração ao cuidado em saúde requer abordagens interdisciplinares que contemplem fatores individuais e sociais, promovendo um atendimento holístico e centrado no paciente.

## Destaques

**(1)** O bem-estar socioemocional é multifacetado e multidimensional. **(2)** O bem-estar socioemocional contribui para a melhoria da qualidade de vida. **(3)** Ter equilíbrio emocional é fundamental para lidar com emoções de forma adaptativa. **(4)** O suporte social contribui diretamente para a resiliência emocional. **(5)** A mensuração do bem-estar socioemocional envolve desafio conceitual e metodológico.

## Introdução

O bem-estar tem ganhado relevância crescente no campo da saúde, especialmente à medida que a atenção ao cuidado se amplia para além de aspectos puramente biológicos e começa a abarcar dimensões emocionais e sociais^1^. Essa perspectiva holística favorece práticas mais humanas e centradas na pessoa, com impacto direto na qualidade do cuidado prestado.

Compreender o bem-estar implica reconhecer que a saúde mental não se restringe à ausência de transtornos, mas envolve equilíbrio emocional, bem-estar psicológico e a capacidade de enfrentar os desafios da vida cotidiana. Assim, ela está intrinsecamente ligada à habilidade de cada indivíduo de lidar com o estresse, trabalhar de forma produtiva e contribuir para sua comunidade^2^ ^-^ ^3^.

A Organização Mundial da Saúde (OMS)^4^ ^)^ define saúde mental como “um estado de bem-estar no qual o indivíduo desenvolve suas habilidades, enfrenta as tensões normais da vida, trabalha de forma produtiva e contribui para sua comunidade”. Esse conceito abrange dimensões emocionais, psicológicas e sociais, influenciando diretamente como as pessoas pensam, sentem, agem, lidam com o estresse, constroem relações interpessoais e tomam decisões saudáveis, sendo estes componentes essenciais e indissociáveis do bem-estar^3^.

Ao longo do tempo, o conceito de bem-estar evoluiu de uma visão restrita à ausência de doença para abordagens mais complexas, como o bem-estar subjetivo e o bem-estar psicológico, que consideram, respectivamente, a satisfação pessoal e a realização plena das potencialidades humanas^3^. Essa evolução evidencia que tanto a vivência de emoções positivas quanto a busca por propósito e crescimento são essenciais para uma vida plena. Contudo, sua complexidade ultrapassa essas dimensões individuais, incorporando também aspectos sociais e relacionais^5^.

Nesse contexto abrangente, o conceito de bem-estar socioemocional emergiu como uma perspectiva integradora, enfatizando a interação entre fatores sociais e emocionais. Esse constructo abrange a capacidade de lidar com desafios diários, desenvolver resiliência e manter relacionamentos saudáveis^6^. Essa perspectiva reflete uma compreensão mais profunda e multifacetada desse fenômeno, ao conceber o bem-estar socioemocional como um equilíbrio entre a regulação emocional, o estabelecimento de relações sociais e o sentido de propósito na vida^7^. Tais abordagens reforçam a compreensão do bem-estar socioemocional como um constructo multidimensional, essencial ao desenvolvimento integral e à qualidade de vida (QV) dos indivíduos.

Apesar do uso crescente do conceito de bem-estar socioemocional na literatura, ainda não há uma definição consensual, o que limita sua aplicação teórica e prática. A complexidade desse constructo exige que pesquisadores e profissionais de saúde considerem experiências subjetivas, influências culturais, sociais e dinâmicas familiares ao desenvolver estratégias de promoção e prevenção em saúde.

Ademais, a variedade de terminologias empregadas na literatura evidencia a necessidade de uma definição uniforme, fundamental para orientar tanto a prática clínica quanto a pesquisa. Nesse contexto, torna-se imprescindível clarificar o conceito de bem-estar socioemocional, o que fundamenta o objetivo deste estudo: analisar o conceito de bem-estar socioemocional no contexto do cuidado em saúde.

## Método

### Tipo de estudo

Estudo teórico de análise de conceito, desenvolvido com base no modelo proposto por Walker e Avant^8^ e operacionalizado mediante revisão de escopo^9^. A análise de conceito desenvolvida é uma abordagem sistemática amplamente utilizada na pesquisa em enfermagem para clarificar conceitos complexos e ampliar sua aplicação na prática clínica^10^.

O modelo^8^ ^)^ compreende oito etapas: 1) seleção do conceito, que consiste na escolha do fenômeno a ser estudado; 2) determinação dos objetivos, quando se definem as finalidades da análise; 3) identificação dos possíveis usos, que explora como o conceito é empregado na literatura; 4) definição dos atributos essenciais, que estabelece as características principais do conceito; 5) identificação de um caso-modelo, que exemplifica o conceito de forma clara e representativa; 6) análise de outros casos, incluindo casos limítrofes, relacionados, contrários, para destacar diferenças e semelhanças; 7) identificação de antecedentes e consequentes, os fatores que precedem e resultam do conceito; e 8) definição de referenciais empíricos, que são indicadores utilizados para medir o conceito na prática.

Para sustentar esta análise, foi incorporada uma revisão de escopo^9^ ^)^ (etapa 3), registrada no *Open Science Framework* (https://osf.io/h5zr6/) sob o DOI 10.17605/OSF.IO/H5ZR6, que subsidiou as etapas subsequentes do modelo.

### Coleta de dados

Para a formulação da questão de pesquisa, utilizou-se o mnemônico PCC: População - Não especificada; Conceito - Bem-estar socioemocional; Contexto - Cuidados de saúde. Com base nessas definições, foi estabelecida a seguinte questão norteadora: Como o conceito de bem-estar socioemocional é abordado no contexto dos cuidados em saúde? As buscas ocorreram em novembro e dezembro de 2024, com a estratégia primária desenvolvida em conjunto com um profissional bibliotecário e ajustada para as oito fontes de dados [PubMed, EMBASE (*Excerpta Medica dataBASE*), *Web of Science*, *Cumulative Index to Nursing and Allied Health Literature* (CINAHL), MEDLINE (*Medical Literature Analysis and Retrieval System Online*), Scopus (Elsevier), Biblioteca Virtual em Saúde (BVS) e *Google* ^*®*^ Acadêmico, combinado com os operadores booleanos AND e OR (Figura 1). 

**Figura 1:** Estratégias de busca. Curitiba, PR, Brasil, 2025

Fonte de dados	Estratégia de busca
BVS*	(“Bem-estar socioemocional” OR “Bienestar socioemocional” OR “Socioemotional well-being” OR “Bem-estar” OR “Bienestar” OR “Well-being”) AND (“Cuidados de saúde” OR “Cuidados de salud” OR “Atenção à saúde” OR “Atención a la salud”)
EMBASE^†^	(‘Socioemotional well-being’:ti,ab,kw OR ‘Well-being’:ti,ab,kw)) AND (‘Health care’:ti,ab,kw OR ‘Health care’/exp OR ‘Healthcare’:ti,ab,kw OR ‘Health services’:ti,ab,kw OR ‘Health services’/exp OR ‘Delivery of health care’:ti,ab,kw OR ‘Delivery of health care’/exp)
MEDLINE^‡^	(‘Socioemotional well-being’:ti,ab,kw OR ‘Well-being’:ti,ab,kw)) AND (‘Health care’:ti,ab,kw OR ‘Health care’/exp OR ‘Healthcare’:ti,ab,kw OR ‘Health services’:ti,ab,kw OR ‘Health services’/exp OR ‘Delivery of health care’:ti,ab,kw OR ‘Delivery of health care’/exp)
PubMed	(“Socioemotional well-being”[Title/Abstract] OR “Well-being”[Title/Abstract]) AND (“Health care”[MeSH Terms] OR “Health care”[Title/Abstract] OR “Healthcare”[Title/Abstract] OR “Health services”[MeSH Terms] OR “Health services”[Title/Abstract] OR “Delivery of health care”[MeSH Terms] OR “Delivery of health care”[Title/Abstract])
Scopus (Elsevier)	TITLE-ABS-KEY((“Socioemotional well-being” OR “Well-being”) AND TITLE-ABS-KEY (“Health care” OR “Healthcare” OR “Health services” OR “Delivery of health care”))
*Web of Science*	ALL=(“Socioemotional well-being” OR “Well-being”) AND style="padding: 10px; border:1px solid black" ALL=(“Health care” OR “Healthcare” OR “Health services” OR “Delivery of health care”)
CINAHL^§^	(“Bem-estar socioemocional” OR “Bienestar socioemocional” OR “Socioemotional well-being” OR “Bem-estar” OR “Bienestar” OR “Well-being”) AND (“Cuidados de saúde” OR “Cuidados de salud” OR “Atenção à saúde” OR “Atención a la salud”)
*Google* ^*®*^ **Acadêmico**	(“Bem-estar socioemocional” OR “Bienestar socioemocional” OR “Socioemotional well-being” OR “Bem-estar” OR “Bienestar” OR “Well-being”) AND (“Cuidados de saúde” OR “Cuidados de salud” OR “Atenção à saúde” OR “Atención a la salud”)

*BVS = Biblioteca Virtual em Saúde; ^†^EMBASE = *Excerpta Medica dataBASE*; ^‡^MEDLINE = *Medical Literature Analysis and Retrieval System Online*; ^§^CINAHL = *Cumulative Index to Nursing and Allied Health Literature*

### Critérios de seleção

Os critérios de inclusão delimitados foram: estudos acessíveis na íntegra, publicados entre 2014 e 2024, período definido para contemplar produções recentes, nos idiomas português, espanhol e inglês; que continham a expressão “bem-estar socioemocional” no título, resumo ou palavras-chave, a garantir a relevância dos artigos com o tema central da pesquisa. Foram excluídos os seguintes estudos: cartas ao editor, editoriais, resumos e estudos de opinião e duplicados.

Todos os artigos identificados foram exportados para o gerenciador de revisões *Rayyan* ^*®*^ . Dois revisores avaliaram, às cegas, títulos, resumos e descritores baseados nos critérios elegíveis. As discordâncias foram resolvidas por um terceiro revisor. Além da seleção nas bases de dados, foi realizada uma busca manual nas listas de referências dos estudos incluídos. 

### Seleção dos estudos, tratamento e análise dos dados

A busca identificou 13.728 estudos, dos quais, 1.883 eram duplicados. Após a triagem de títulos, resumos e palavras-chave relacionados ao termo bem-estar socioemocional, 11.785 foram excluídos. Restaram 60 estudos para leitura completa, dos quais 53 foram descartados por não atenderem aos critérios de inclusão. Foram considerados apenas os artigos que abordaram explicitamente o conceito de bem-estar socioemocional, resultando em sete estudos incluídos na revisão final.

Após a seleção, os estudos foram organizados na seguinte disposição: autores e ano, pais, população e amostra, objetivo principal, conceito do bem-estar socioemocional no contexto da saúde e dimensões observadas, conforme ilustrado na Figura 2. A definição dos atributos essenciais do bem-estar socioemocional se deu por meio da seguinte questão: *Quais são as características fundamentais que definem o bem-estar socioemocional?* Para identificação dos seus antecedentes, formulou-se a pergunta: *Quais eventos precedem o bem-estar socioemocional?* Por fim, para determinar seus consequentes, considerou-se a questão: *Quais eventos resultam do bem-estar socioemocional?*

**Figure 2:** Characterization of selected studies. Curitiba, PR, Brazil, 2025

Autores e ano	País	População e amostra	Objetivo principal	Conceito do bem-estar socioemocional	Dimensões observadas
Ashton et al., 2024^11^	Nova Zelândia	Pais (n=257) e filhos (n=285)	Avaliar o impacto a longo prazo (12 meses) do programa ministrado na comunidade	Efeito positivo das atividades físicas comunitárias no bem-estar socioemocional	Fortalecimento de vínculos familiares; redução do estresse; suporte emocional; ambiente positivo
Tunalliar et al., 2024^12^	EUA*	Provedores (2.027) de Lar Adotivos para Adultos	Monitorar mudanças no envolvimento familiar após a pandemia de COVID-19	Efeito da redução do envolvimento familiar no bem-estar socioemocional	Dependência do envolvimento familiar; solidão; estresse; continuidade dos vínculos afetivos
Us et al., 2024^1^	Turquia	Crianças (n=600) de profissionais de saúde	Explorar problemas socioemocionais e comportamentais	Impacto emocional associado ao estresse familiar e social	Vulnerabilidade infantil; impacto do estresse; influência do trabalho materno
Timmer et al., 2022^13^	Austrália, Suíça, EUA e Reino Unido	Revisão	Recomendações para inclusão do bem-estar socioemocional na reabilitação audiológica	Impacto da perda auditiva no bem-estar socioemocional	Autoestima reduzida; exclusão social; ansiedade; depressão; apoio emocional
Siette et al., 2021^14^	Austrália	Idosos (n=21) em assistência domiciliar	Impacto da pandemia na qualidade de vida e redes sociais	Bem-estar socioemocional afetado pelo isolamento social	Isolamento; solidão; depressão; importância das redes de apoio
Fenwick et al., 2020^15^	Cingapura e Austrália	Adultos (n=220) com retinopatia diabética	Validar índice de utilidade da retinopatia diabética	Impacto da perda de visão no bem-estar socioemocional	Autoestima; relações sociais; percepção de perda; limitações; impacto da doença crônica
Pruner et al., 2020^16^	EUA*	Pais adotivos/biológicos (n=25) de crianças com ou em risco de TEAF	Identificar características da intervenção precoce	Bem-estar socioemocional dos pais	Equilíbrio emocional; dinâmica familiar; suporte; enfrentamento; redução de estresse

*USA = United States of America, †UK = United Kingdom; ‡FASD = Fetal Alcohol Spectrum Disorders

### Uso de inteligência artificial

Os autores declaram a utilização do ChatGPT(https://chatgpt.com) para a elaboração da Figura 5, que apresenta um diagrama conceitual ilustrando as relações entre fatores associados ao bem-estar socioemocional. Os dados e a figura passaram por edição dos autores para aprimoramento e melhorias.

## Resultados

A apresentação dos resultados está de acordo com as etapas de análise de conceito segundo Walker e Avant^8^.

### Seleção e objetivo

“Bem-estar” é frequentemente definido como um estado de equilíbrio físico, mental e social, no qual o indivíduo se sente saudável, confortável e em harmonia consigo mesmo e com o ambiente. Esse estado envolve aspectos emocionais e psicológicos, como felicidade, segurança e realização, além de componentes físicos, como saúde e vitalidade^5^.

Adicionalmente, o bem-estar pode se estender ao contexto social, abrangendo relações interpessoais saudáveis e um senso de pertencimento à comunidade. De forma geral, ele representa a condição de estar bem de maneira holística, incluindo a ausência de sofrimento, uma boa QV e a capacidade de enfrentar desafios de maneira adaptativa e positiva^5^.

Embora a OMS não forneça uma definição específica para “bem-estar socioemocional”, o conceito parece estar intimamente relacionado à saúde mental, pois envolve a capacidade de lidar com emoções, estabelecer relações interpessoais saudáveis e manter um equilíbrio emocional. De acordo com o *Australian Institute of Health and Welfare* ^17^, o bem-estar socioemocional é descrito como parte essencial do bem-estar mental e social, que vai além da ausência de doença e inclui a capacidade de manter relacionamentos positivos, gerenciar emoções e participar ativamente na comunidade, abrangendo resiliência emocional, senso de pertencimento e suporte social.

A falta de consenso nas definições do bem-estar socioemocional evidencia sua complexidade e reforça a necessidade de uma conceituação clara e contextualizada. Essa ausência de consenso limita sua aplicação em pesquisas e intervenções, dificultando comparações e generalizações. Estabelecer uma definição consistente, flexível e aplicável a diferentes contextos é essencial para consolidar o conceito, orientar práticas eficazes de cuidado e promover o bem-estar socioemocional de forma ampla e significativa.

### Identificação dos possíveis usos do conceito

Este tópico identifica os possíveis usos do conceito de bem-estar, com base nos sete estudos selecionados na etapa três, que exploram suas aplicações, características e implicações práticas em diferentes contextos.

Um estudo conduzido^1^ ^)^ na Turquia utilizou o conceito de bem-estar socioemocional para investigar como o estresse e a sobrecarga de trabalho de mães profissionais de saúde, durante a pandemia de COVID-19, afetaram o equilíbrio emocional e comportamental de suas crianças. O estudo destacou que o bem-estar socioemocional das crianças foi comprometido, resultando em maiores taxas de ansiedade, depressão e problemas comportamentais. Desse modo, o bem-estar socioemocional foi aplicado para entender como fatores externos, como o estresse parental, impactam a saúde mental infantil e reforçam a necessidade de políticas de apoio às famílias em situações de crise.

Em um contexto diferente, um estudo^15^ realizado em Cingapura e Austrália abordou indiretamente o bem-estar socioemocional ao validar um índice de utilidade para pacientes com retinopatia diabética. Embora o foco principal fosse a QV relacionada à saúde, o estudo destacou que a perda de visão pode levar ao isolamento social e à depressão, impactando o bem-estar socioemocional dos pacientes. Nesse caso, o conceito foi utilizado para enfatizar a importância de considerar aspectos emocionais e sociais no tratamento de condições crônicas.

Nos Estados Unidos da América (EUA), pesquisadores^16^ ^)^ exploraram as experiências de pais de crianças com transtorno do espectro alcoólico fetal (TEAF), utilizando o conceito de bem-estar socioemocional para entender como o estresse e a sobrecarga emocional afetam a saúde mental dos pais. O estudo revelou que muitos pais enfrentam sentimento de culpa, estresse e isolamento, comprometendo seu equilíbrio emocional. Aqui, o conceito foi aplicado para destacar a necessidade de suporte psicológico e prático para os pais, promovendo um ambiente familiar mais saudável.

Na Austrália^14^, investigou-se o impacto da pandemia de COVID-19 na QV de idosos que recebem cuidados comunitários, utilizando o conceito de bem-estar socioemocional para explorar como o isolamento social e a interrupção de serviços afetaram a saúde mental dos idosos. O estudo mostrou que muitos idosos relataram solidão e depressão, destacando a importância de estratégias para manter o contato social e o suporte emocional. Nesse contexto, o conceito foi aplicado para enfatizar a necessidade de abordagens integradas que considerem tanto o cuidado físico quanto o emocional.

Outro estudo^13^, colaboração entre Austrália, Suíça, EUA e Reino Unido, focou o bem-estar socioemocional de adultos com perda auditiva. O conceito foi utilizado para discutir como a perda auditiva pode levar ao isolamento social e à depressão, impactando a QV. O estudo recomendou a inclusão de estratégias para promover o bem-estar socioemocional, como aconselhamento psicológico e suporte social, nos programas de reabilitação audiológica. O conceito foi aplicado para destacar a importância de uma abordagem holística no tratamento de condições crônicas.

Nos EUA^12^, analisou-se o envolvimento familiar entre residentes adultos de lares adotivos, utilizando o conceito de bem-estar socioemocional para explorar como a redução do contato familiar durante a pandemia afetou a saúde mental dos residentes. O estudo mostrou que muitos residentes relataram sentimentos de abandono e solidão, destacando a importância do envolvimento familiar para o equilíbrio emocional. Nesse caso, o conceito foi aplicado para reforçar a necessidade de políticas que fortaleçam os laços familiares e promovam o bem-estar socioemocional.

Na Nova Zelândia^11^, avaliaram-se os resultados de um programa comunitário de atividade física entre pais e filhas, utilizando o conceito de bem-estar socioemocional para explorar como a interação social e a atividade física impactam a saúde mental e emocional dos participantes. O estudo mostrou que o programa promoveu melhorias significativas no bem-estar socioemocional, fortalecendo os vínculos familiares e reduzindo o estresse. O conceito foi aplicado para destacar a importância de programas que integram atividade física e interação social para promover a saúde mental.

Ao analisar esses estudos, reforça-se que o bem-estar socioemocional é um conceito multidimensional, essencial para a QV em diferentes fases e contextos. Desde o impacto da pandemia na saúde mental infantil até os desafios enfrentados por idosos e adultos com condições crônicas, o equilíbrio emocional e social emerge como um fator crucial para o desenvolvimento humano.

### Atributos essenciais

A definição dos atributos essenciais é uma etapa fundamental na análise de conceito, pois eles são características que esclarecem a essência do conceito, permitindo reconhecê-lo, diferenciá-lo e validar sua aplicabilidade em diferentes contextos^8^. A análise dos estudos selecionados revelou os atributos essenciais do bem-estar socioemocional.

#### Equilíbrio emocional

O equilíbrio emocional refere-se à capacidade de lidar com emoções de maneira estável e adaptativa, minimizando impactos negativos da ansiedade, do estresse e da tristeza. Ele é essencial para a manutenção da saúde mental e influencia diretamente a forma como os indivíduos enfrentam desafios cotidianos^14^ ^,^ ^16^.

A relevância do equilíbrio emocional fica evidente em diferentes contextos. Uma pesquisa^14^ que acompanhou idosos em cuidados comunitários durante a pandemia utilizou escalas de saúde mental e entrevistas semiestruturadas para analisar a relação entre equilíbrio emocional e QV. Os resultados indicaram que idosos com maior equilíbrio emocional apresentavam menores níveis de solidão e depressão, demonstrando a relevância desse atributo para o bem-estar socioemocional, especialmente em situações de vulnerabilidade.

Para além do impacto individual, o equilíbrio emocional também influencia o ambiente familiar. Estudo^16^ sobre pais de crianças com ou em risco de TEAF identificou que o equilíbrio emocional dos pais impacta diretamente o bem-estar das famílias. Pais com maior controle emocional apresentaram melhor adaptação às dificuldades enfrentadas, contribuindo para a saúde emocional de todos os membros da família. Esses achados reforçam que o equilíbrio emocional fortalece o indivíduo e desempenha um papel crucial nas relações interpessoais e na dinâmica familiar.

#### Percepção de suporte social

A percepção de suporte social relaciona-se ao sentimento de apoio recebido de familiares, amigos e comunidades. Desempenha um papel central na resiliência emocional e no enfrentamento de dificuldades, promovendo senso de pertencimento e segurança. Além disso, sentir-se apoiado está diretamente associado à confiança e à estabilidade emocional^11^ ^,^ ^13^.

A percepção de suporte social é um fator determinante para o bem-estar emocional, especialmente em grupos vulneráveis. Em pacientes com perda auditiva, sentir-se apoiado reduziu os níveis de estresse e facilitou a adaptação às limitações, conforme demonstrado por uma pesquisa que utilizou questionários e testes psicológicos^13^. No contexto familiar, o suporte foi essencial para o bem-estar socioemocional de residentes em lares adotivos, enquanto sua ausência, especialmente durante a pandemia de COVID-19, esteve associada a maior estresse e dificuldades emocionais^12^.

Além do ambiente familiar, o suporte da comunidade também exerce um papel fundamental. Um estudo que analisou a participação de pais e filhos em um programa de atividade física comunitária demonstrou que o apoio social fortaleceu os laços familiares e promoveu o sentimento de pertencimento, contribuindo para o bem-estar socioemocional dos participantes^11^.

#### Sensação de controle sobre a própria vida

A sensação de controle sobre a própria vida está relacionada à percepção de autonomia e à capacidade de tomar decisões que influenciam o próprio bem-estar. Esse atributo está fortemente associado à autoconfiança, independência e satisfação pessoal^15^.

A percepção de controle sobre a vida tem demonstrado um impacto significativo na adaptação a condições adversas. Um estudo^15^ com pacientes com retinopatia diabética, conduzido por meio de experimentos de escolha discreta e relatos de QV, observou que aqueles que se sentiam no comando das próprias decisões de saúde relataram maior satisfação e uma adaptação mais positiva à condição crônica. Isso evidencia a importância de se sentir no controle para o bem-estar emocional e a resiliência diante de condições de saúde complicadas.

Em um contexto semelhante, a pesquisa^14^ realizada com idosos em cuidados comunitários durante a pandemia de covid-19 destacou a relevância da autonomia na saúde emocional. Aqueles que mantiveram uma percepção de controle sobre suas vidas apresentaram menos ansiedade e melhor QV, sugerindo que a capacidade de gerenciar a própria vida, especialmente em momentos de grande incerteza, é fundamental para a manutenção da saúde emocional.

#### Habilidades de regulação emocional

As habilidades de regulação emocional envolvem a capacidade de reconhecer, compreender e gerenciar emoções de forma adaptativa, permitindo que os indivíduos enfrentem desafios sem desenvolver respostas desproporcionais de estresse ou ansiedade^13^.

A relevância desse atributo foi evidenciada em diferentes populações. Uma investigação^16^ com pais de crianças com TEAF, baseada em entrevistas e escalas de regulação emocional, apontou que aqueles com maior domínio dessas habilidades apresentaram melhor adaptação a desafios complexos, favorecendo o equilíbrio psicológico e a resiliência.

Além disso, uma revisão^13^ sobre o impacto da perda auditiva no bem-estar socioemocional de adultos destacou que a regulação emocional desempenha um papel essencial na adaptação à perda auditiva. Pacientes que demonstraram maior capacidade de gerenciar suas emoções enfrentaram os desafios da condição de maneira mais positiva, evidenciando que o desenvolvimento dessas habilidades pode mitigar os impactos negativos de limitações físicas sobre a QV. A Figura 3 mostra os atributos essenciais elencados. 

**Figura 3:** Atributos essenciais do bem-estar socioemocional. Curitiba, PR, Brasil, 2025

Atributos	Descrição	Estudos relacionados
Equilíbrio emocional	Capacidade de gerenciar emoções.	Siette, et al. (2021): Idosos que mantiveram equilíbrio emocional relataram menor solidão e depressão^14^.
Percepção de suporte social	Sensação de pertencimento e conexão.	Timmer, et al. (2022): Pacientes com perda auditiva que percebiam suporte social tiveram melhor bem-estar emocional^13^.
Sensação de controle sobre a própria vida	Autonomia e capacidade de tomar decisões.	Fenwick, et al. (2020): Pacientes que sentiam controle sobre sua condição relataram maior qualidade de vida^15^.
Habilidades de regulação emocional	Capacidade de identificar e regular emoções.	Pruner, et al. (2020): Pais que desenvolveram habilidades de regulação emocional lidaram melhor com o estresse^16^.

Esses atributos são fundamentais para o bem-estar socioemocional e se manifestam em diferentes contextos. A percepção de suporte social fortalece o senso de pertencimento e conexão, enquanto a sensação de controle sobre a própria vida promove autonomia e reduz a ansiedade. O equilíbrio emocional permite lidar com estressores de forma adaptativa, impactando positivamente a saúde mental. Além disso, as habilidades de regulação emocional capacitam os indivíduos a gerenciar suas emoções de maneira eficaz, prevenindo reações desproporcionais e promovendo o bem-estar geral.

### Caso modelo

Os casos são exemplos usados para ilustrar e esclarecer o conceito em estudo. Eles ajudam a diferenciar o conceito dos termos relacionados e a mostrar como ele pode ser aplicado em diferentes contextos^8^.

O caso modelo representa a manifestação completa do conceito de bem-estar socioemocional. L.P.M. 45 anos, professora universitária recentemente diagnosticada com câncer de mama em estágio inicial, desde o diagnóstico apresenta crises de ansiedade que se intensificam no período noturno. Iniciou acompanhamento profissional com psicoterapia, prática regular de meditação guiada e participação em grupo comunitário de artes da comunidade. Ao longo de oito meses, desenvolveu atributos essenciais do bem-estar socioemocional. L.P.M. demonstra capacidade de lidar com situações complexas associadas ao tratamento, mantém vínculos significativos com familiares e colegas, percebe-se apoiada em sua rede social e expressa sentimentos de esperança em relação ao futuro. Além disso, apresenta uma regulação saudável das emoções diante de situações estressoras, consegue ressignificar sua experiência de adoecimento e mantém um nível satisfatório de engajamento em atividades cotidianas. Como resultado, apresenta redução de angústias emocionais, melhor desempenho nas atividades cotidianas e maior QV. Esse caso reflete a manifestação integral do conceito de bem-estar socioemocional.

### Análise de outros casos

#### Caso limítrofe

O caso limítrofe contém alguns, mas não todos os atributos essenciais do conceito^8^. L.G.P., 42 anos, com diagnóstico de depressão desencadeada por estressores laborais, iniciou acompanhamento com terapia cognitivo-comportamental e tratamento medicamentoso. Como complemento, passou a realizar atividades físicas regulares e a dedicar-se a práticas de lazer que lhe proporcionam prazer. Apesar da melhora inicial, com enfrentamento mais positivo e sensação de maior controle sobre a própria vida, ainda apresenta instabilidade emocional (humor deprimido, irritabilidade, ansiedade) e dificuldades na regulação emocional, especialmente diante de demandas relacionadas ao trabalho. Embora os sintomas depressivos tenham diminuído, persistem oscilações psicológicas significativas. Trata-se, portanto, de um caso limítrofe, em que alguns atributos do bem-estar socioemocional estão presentes, mas não de forma integral. 

#### Caso contrário

O caso contrário exemplifica o “não conceito”, ou seja, a ausência total ou quase total dos atributos essenciais do conceito^8^. C.O.R., 38 anos, recém-mudado do interior para São Paulo-SP, apresenta transtornos de ansiedade e depressão. Embora tenha se consultado com um psiquiatra, não seguiu as recomendações médicas nem buscou acompanhamento psicológico ou terapêutico adicional. Manifesta episódios recorrentes de irritabilidade, humor deprimido, ansiedade e sensação de perda de controle. Vive isolado e sem redes de apoio, permanecendo grande parte do tempo recluso em seu quarto, sem engajamento em atividades sociais ou de lazer. Nesse contexto, os sintomas de ansiedade e depressão são agravados pela ausência de suporte social e estratégias adequadas de enfrentamento. Evidencia-se, assim, baixa QV, com ausência dos atributos essenciais do bem-estar socioemocional.

#### Caso relacionado

O caso relacionado ilustra uma situação que está associada ao conceito de bem-estar socioemocional^8^. G.T., 28 anos, estudante do sexto ano de medicina, apresenta estresse crônico, insônia e instabilidade emocional, durante grande parte do período letivo. Acredita estar com síndrome ansiosa e depressiva. Contudo, ao término da temporada de provas, observa-se uma melhora significativa do quadro, devido à diminuição do estresse e à retomada de atividades físicas, práticas de lazer e convivência social com amigos. Este caso está relacionado ao conceito, mas não contempla integralmente seus atributos essenciais, pois não há procura por avaliação profissional e acompanhamento terapêutico, essenciais para o desenvolvimento completo do bem-estar socioemocional.

### Antecedentes e consequentes

Os antecedentes referem-se às causas ou circunstâncias que precedem a promoção do bem-estar socioemocional^8^. A análise dos estudos revelou que diversos fatores contribuem para o desenvolvimento desse conceito.

#### Resiliência

A capacidade de superar adversidades e se adaptar a circunstâncias desafiadoras desempenha um papel essencial no equilíbrio emocional e na manutenção do bem-estar socioemocional^15^. Indivíduos mais resilientes tendem a apresentar maior controle sobre a própria vida e a desenvolver estratégias mais eficazes para lidar com situações estressantes, o que favorece sua adaptação a diferentes contextos e contribui para melhoria da QV^15^.

#### Suporte social

O apoio emocional e prático oferecido por familiares, amigos ou redes comunitárias tem um impacto direto na redução do estresse e no fortalecimento do bem-estar. A presença de uma rede de suporte contribui para o enfrentamento de desafios e promove um senso de pertencimento, favorecendo a estabilidade emocional^14^. Além disso, ambientes que incentivam a interação social fortalecem os vínculos interpessoais e criam condições propícias para o desenvolvimento socioemocional saudável^13^.

#### Autoconhecimento

Pessoas com maior grau de autoconhecimento lidam melhor com desafios diários, evitando respostas impulsivas ou desproporcionais. Esse fator está diretamente ligado ao desenvolvimento da resiliência e ao fortalecimento das habilidades emocionais, contribuindo para uma melhor adaptação a diferentes circunstâncias da vida^16^.

#### Ambiente favorável

Um contexto que oferece segurança emocional, apoio social e acesso a recursos adequados influencia diretamente o bem-estar socioemocional. Ambientes acolhedores promovem maior estabilidade emocional e facilitam a criação de relações interpessoais positivas. A presença de espaços que estimulam interações saudáveis e proporcionam suporte contínuo ajuda a fortalecer a regulação emocional e a capacidade de enfrentamento de desafios^16^.

#### Estabilidade emocional

Manter um estado emocional equilibrado diante de situações adversas é um fator essencial para o bem-estar socioemocional. Indivíduos emocionalmente estáveis apresentam maior controle sobre suas ações e reações, o que favorece uma adaptação mais positiva a diferentes circunstâncias^15^. Essa estabilidade está relacionada à capacidade de regular emoções e enfrentar desafios de maneira racional e resiliente.

#### Autonomia e tomada de decisão

A percepção de controle sobre a própria vida está diretamente associada ao bem-estar socioemocional. Ter autonomia para tomar decisões, especialmente no gerenciamento da própria saúde e na organização da vida cotidiana, reduz níveis de ansiedade e promove maior satisfação pessoal. Sentir-se no comando das próprias escolhas fortalece a autoconfiança e proporciona um maior senso de realização e propósito^15^.

#### Vínculos interpessoais positivos

Relacionamentos saudáveis e interações sociais significativas são fundamentais para o bem-estar socioemocional. Laços interpessoais baseados em confiança e apoio mútuo oferecem segurança emocional e contribuem para a construção de um senso de pertencimentos^14^. Ambientes que incentivam essas conexões fortalecem a percepção de suporte social e promovem maior estabilidade emocional ao longo do tempo.

Os consequentes do bem-estar socioemocional referem-se aos resultados que surgem como consequência desse estado^8^. 

#### Melhora na qualidade de vida

O bem-estar socioemocional contribui para um estado geral mais equilibrado, refletindo-se diretamente na QV. Indivíduos que apresentam maior estabilidade emocional e suporte social adequado enfrentam desafios com mais resiliência, demonstram maior satisfação pessoal e mantêm uma visão mais positiva da vida. Isso é perceptível na adaptação a condições crônicas de saúde, no enfrentamento de dificuldades familiares e no gerenciamento do estresse do dia a dia^14^.

#### Aumento do engajamento social

Pessoas que experimentam um estado emocional equilibrado tendem a buscar e manter conexões sociais de maneira mais ativa, participando de atividades comunitárias, estabelecendo laços mais sólidos com familiares e amigos e desenvolvendo um senso de pertencimento. Essa interação favorece a troca de apoio e cria um ciclo virtuoso no qual o contato social reforça o bem-estar emocional^11^.

#### Redução de sintomas psicossociais

A capacidade de regular emoções de maneira eficaz e a presença de uma rede de suporte confiável permitem que os indivíduos lidem melhor com situações adversas, minimizando impactos negativos à saúde mental. Em grupos vulneráveis, como idosos, doentes crônicos ou indivíduos que enfrentam mudanças significativas em suas vidas, a presença do bem-estar socioemocional pode ser um fator determinante para a prevenção de transtornos psicológicos^13^.

#### Melhor desempenho em atividades cotidianas

Indivíduos com maior equilíbrio emocional demonstram maior motivação, concentração e capacidade de adaptação, o que se reflete em um melhor rendimento no ambiente escolar, no trabalho e nas interações familiares. Crianças e adultos com elevado bem-estar socioemocional apresentam maior envolvimento em suas tarefas e uma abordagem mais positiva diante de desafios, favorecendo um funcionamento mais produtivo e eficiente^1^.

Os consequentes do bem-estar socioemocional evidenciam seus amplos benefícios, abrangendo aspectos emocionais, sociais e funcionais. A estabilidade psicológica favorece a participação ativa na vida social e a manutenção de uma boa QV, criando um ciclo positivo no qual indivíduos emocionalmente equilibrados tendem a desenvolver relações mais saudáveis e a enfrentar desafios com mais segurança. Dessa forma, promover e fortalecer o bem-estar socioemocional se torna uma estratégia essencial para melhorar a saúde mental e o bem-estar geral da população. 

Na Figura 4, estão apresentados os antecedentes e consequentes do bem-estar socioemocional.

**Figura 4:** Antecedentes e consequentes do bem-estar socioemocional. Curitiba, PR, Brasil, 2025

Categoria	Elementos	Descrição	Estudos relacionados
Antecedentes	Resiliência	Lidar com adversidades, como estresse parental na pandemia.	Us, et al. (2024): Resiliência mitigou efeitos do estresse em crianças de mães profissionais de saúde^1^.
	Suporte social	Redes de apoio promovem estabilidade emocional.	Tunalilar, et al. (2024): Apoio familiar essencial para bem-estar em lares adotivos para adultos^12^.
	Autoconhecimento	Consciência emocional auxilia na regulação do estresse.	Pruner, et al. (2020): Pais de crianças com transtorno do espectro alcoólico fetal destacaram a importância do autoconhecimento^16^.
	Ambiente favorável	Contextos seguros e com recursos fortalecem o bem-estar socioemocional.	Ashton, et al. (2024): Programas comunitários promovem ambiente positivo^11^.
	Estabilidade emocional	Capacidade de manter o equilíbrio diante de emoções.	Fenwick, et al. (2020): Regulação emocional em situações adversas favorece a adaptação positiva em diferentes circunstâncias^15^.
	Autonomia	Controle sobre decisões aumenta a confiança e propósito.	Fenwick, et al. (2020): Autonomia fortalece autoestima e satisfação^15^.
	Vínculos interpessoais positivos	Relações saudáveis e de apoio que contribuem para o bem-estar socioemocional.	Siette, et al. (2021): Idosos com suporte e interações sociais significativas possuem maior estabilidade emocional ao longo do tempo^14^.
Consequentes	Melhora na qualidade de vida	Maior satisfação e bem-estar.	Siette, et al. (2021): Idosos com suporte tiveram melhor qualidade de vida^14^.
	Engajamento social	Aumento da interação e participação social.	Ashton, et al. (2024): Programas de atividade estimularam maior engajamento^11^.
	Redução de sintomas psicológicos	Menos ansiedade e depressão.	Timmer, et al. (2022): Pacientes com apoio relataram melhora emocional^13^.
	Melhor desempenho	Maior produtividade e funcionalidade.	Us, et al. (2024): Crianças com maior bem-estar tiveram melhor desempenho escolar^1^.

A Figura 5 apresenta um diagrama conceitual que ilustra as relações entre fatores associados ao bem-estar socioemocional, organizados em três níveis: antecedentes, atributos e consequentes.

**Figura 5:** Diagrama conceitual do bem-estar socioemocional. Curitiba, PR, Brasil, 2025

Fonte: OpenAI *QV = Qualidade de vida

## Discussão

### Definição de referenciais empíricos

Os referenciais empíricos são as ferramentas de medição que demonstram a ocorrência do conceito, requerendo a identificação de indicadores observáveis, métodos de mensuração e padrões de manifestação^8^. 

Os estudos analisados fornecem evidências sobre como o bem-estar socioemocional se manifesta e é influenciado por fatores externos, permitindo uma compreensão mais estruturada de suas características fundamentais. No entanto, sua mensuração ainda enfrenta desafios conceituais e metodológicos, pois não há um instrumento que capture integralmente a complexidade das experiências associadas a esse conceito.

A saúde mental e o bem-estar são constructos multideterminados, moldados por fatores biológicos, sociais e culturais que influenciam a percepção, a vivência emocional e as estratégias de enfrentamento e interação social dos indivíduos. Dado que cada contexto cultural define parâmetros próprios para o que constitui saúde mental, a sensibilidade a essas nuances é imperativa para a relevância e a eficácia de qualquer intervenção. Essa complexidade inerente demanda o desenvolvimento de instrumentos avaliativos capazes de apreender suas múltiplas dimensões, viabilizando uma mensuração abrangente e devidamente contextualizada do conceito^2^.

Os estudos revisados utilizaram instrumentos padronizados para avaliar diferentes dimensões do bem-estar socioemocional, incluindo QV, apoio social e bem-estar emocional. A Escala de Rede Social de Lubben (LSNS-6) foi empregada para medir o apoio social percebido, com foco nas redes de apoio familiar e de amigos. Já o EQ-5D-5L (EuroQoL-5D-5L) avaliou a qualidade de vida relacionada à saúde(QVRS) em cinco dimensões: mobilidade, autocuidado, dor/desconforto, atividades habituais e sintomas de ansiedade/depressão^14^.

Outros instrumentos foram aplicados para mensurar aspectos específicos do bem-estar socioemocional em diferentes populações. A Escala de Avaliação Social e Emocional Breve (BITISEA), por exemplo, foi utilizada para avaliar a saúde social e emocional de crianças de até três anos de idade^1^. No contexto da assistência auditiva, questionários como o *Patient Health Questionnaire*-4 (PHQ-4), o Questionário de Perda Auditiva de Aceitação e Ação, o Questionário de Restrições de Participação Social (SPARQ) e sua versão reduzida de cinco itens (SIM), além do Inventário de *Handicap* Auditivo para Idosos (HHIE-S), foram recomendados para investigar o impacto da perda auditiva no bem-estar socioemocional^13^.

Além disso, um estudo multicêntrico^15^ incorporou o bem-estar socioemocional ao desenvolvimento do *Disease-Related Utility Index* (DRU-I), uma escala voltada para a avaliação da QV relacionada à audição. Nessa escala, o bem-estar socioemocional foi considerado uma das cinco dimensões centrais da QV dos pacientes. No entanto, a complexidade desse constructo exige uma abordagem mais aprofundada, que contemple suas múltiplas dimensões e os fatores que o influenciam, permitindo uma avaliação mais abrangente e precisa.

É importante destacar que nenhum dos estudos revisados aplicou ou desenvolveu uma escala que meça objetivamente o bem-estar socioemocional. As ferramentas utilizadas avaliam aspectos correlacionados, como sintomas de depressão, ansiedade e satisfação com a vida, mas não capturam o constructo de forma abrangente e específica. Isso evidencia uma lacuna na literatura quanto à mensuração direta do bem-estar socioemocional.

A análise dos estudos revisados revela que o bem-estar socioemocional é um constructo multidimensional, influenciado por diversos fatores, como a adaptação ao estresse^12^, a qualidade das relações interpessoais^15^ e o apoio social^14^. Além disso, conceitos como resiliência emocional^16^, autoestima^13^, suporte familiar^11^ e adaptação social^1^ são fundamentais para promover o bem-estar socioemocional, especialmente em contextos adversos, como crises de saúde pública e doenças crônicas. Esses fatores têm um impacto significativo na saúde mental de indivíduos de diferentes faixas etárias e são essenciais para a elaboração de intervenções mais eficazes e personalizadas. 

Assim, é crucial que as estratégias de promoção do bem-estar socioemocional sejam integradas às práticas de cuidados de saúde e políticas públicas, reconhecendo a complexidade e a interdependência dos fatores que sustentam a saúde mental e emocional ao longo da vida^18^. As intervenções devem ser adaptadas às necessidades específicas de cada população, garantindo que os aspectos emocionais sejam efetivamente incorporados às abordagens voltadas para a saúde e QV.

Um ponto central que emerge da análise é que, embora diferentes contextos em que possa ser investigado, como cuidados paliativos^19^, dietas terapêuticas^20^, doenças crônicas^21^ ou experiências de minorias culturais^22^ ^-^ ^24^, em todos os casos o bem-estar socioemocional aparece como um mediador da adaptação ao adoecimento e às adversidades. Isso reforça a ideia de que a saúde não pode ser compreendida apenas em termos biomédicos, mas também deve ser entendida como um fenômeno que integra processos emocionais, relações sociais e condições estruturais de vida.

Outro aspecto relevante é a dimensão relacional do bem-estar socioemocional. Estudos realizados tanto em populações indígenas^22^ ^-^ ^24^ quanto em contextos clínicos^19^ revelam que a qualidade das conexões sociais, o apoio comunitário e a valorização cultural desempenham um papel central na preservação do bem-estar socioemocional. Essa perspectiva amplia a compreensão do constructo, afastando-o de uma leitura individualizada e aproximando-o de uma visão ecossistêmica, na qual família, comunidade e cultura são determinantes.

A discussão também aponta para a importância da equidade em saúde como fator determinante do bem-estar socioemocional. Estudos^21^ ^,^ ^25^ realizados durante a pandemia de COVID-19 evidenciam como determinantes sociais, como renda, acesso a serviços e suporte social, afetam diretamente o bem-estar socioemocional. Esses achados reforçam que as políticas públicas precisam considerar que a vulnerabilidade social se traduz não apenas em pior desfecho clínico, mas também em maior sofrimento emocional e social, reforçando a interdependência entre desigualdade estrutural e saúde mental.

Nesse contexto, é fundamental reconhecer que o conceito de bem-estar varia significativamente entre indivíduos e comunidades, moldado por fatores como contexto sociocultural, experiências pessoais e valores coletivos. Longe de ser um conceito estático ou universal, o bem-estar se configura como uma construção dinâmica e situada, cuja compreensão exige a integração de múltiplas perspectivas. Essa variabilidade semântica sublinha a importância de abordagens sensíveis às particularidades de cada grupo^26^.

Cabe destacar que os estudos analisados revelam que o bem-estar socioemocional deve ser compreendido como um constructo multidimensional e dinâmico, atravessado por experiências de vida, contextos culturais e condições sociais^3^. Ao mesmo tempo, essas análises apontam para uma lacuna importante: a ausência de instrumentos específicos que mensurem de forma direta essa dimensão. Essa limitação metodológica não invalida os avanços teóricos, mas indica a necessidade de desenvolver medidas mais abrangentes, que captem a complexidade do constructo e sirvam de base para práticas de cuidado e políticas públicas orientadas para a promoção da saúde integral^20^ ^,^ ^24^ ^-^ ^25^.

Uma limitação desta análise de conceito reside na falta de padronização terminológica e conceitual nos estudos sobre o bem-estar socioemocional. A diversidade de definições e terminologias empregadas dificulta a comparação de resultados entre diferentes pesquisas, comprometendo a construção de uma base conceitual coesa. Além disso, o recorte temporal adotado pode ter excluído pesquisas relevantes publicadas fora do período analisado, limitando a abrangência da revisão. Da mesma forma, a utilização apenas do *Google* ^*®*^ Acadêmico como fonte de literatura cinzenta pode ter limitado o acesso a perspectivas complementares. 

## Conclusão

O bem-estar socioemocional é um constructo multidimensional, que vai além da saúde mental individual, englobando interações sociais, emocionais e culturais. Fatores como suporte social, relações interpessoais, contexto sociocultural e condições de vida influenciam diretamente sua manifestação e percepção pelos indivíduos, destacando a necessidade de compreender o conceito de forma integrada e contextualizada.

A análise de conceito realizada contribui para consolidar o entendimento do bem-estar socioemocional, oferecendo suporte para a elaboração de políticas públicas e intervenções de cuidado que integrem aspectos emocionais, sociais e culturais. Ao reconhecer a variabilidade do conceito entre diferentes contextos e populações, torna-se possível promover estratégias mais eficazes voltadas à saúde integral e à QV.

## Data Availability

Todos os dados gerados ou analisados durante este estudo estão incluídos neste artigo publicado.

# Translation: Bienestar socioemocional en el contexto de cuidado en salud: un análisis de concepto^*^

**DOI:** 10.1590/1518-8345.8071.4797

**Authors:** Cristiano de Oliveira Ribeiro, Luciana Puchalski Kalinke, Nuno Filipe de Oliveira Gil Salgado, Luciana de Alcantara Nogueira, Daniela Lourenço Pinto, Tereza Maria Mendes Diniz de Andrade Barroso

**Affiliations:** 1 Universidade Federal do Paraná, Campus Jardim Botânico, Curitiba, PR, Brasil.; 2 Becario de la Coordenação de Aperfeiçoamento de Pessoal de Nível Superior (CAPES), Brasil.; 3 Escola Superior de Enfermagem de Coimbra, Unidade de Investigação, Coimbra, CO, Portugal.

**Keywords:** Salud Mental, Calidad de Vida, Atención a la Salud, Formación de Concepto, Satisfacción Personal, Atención Dirigida al Paciente

## Abstract

**Objetivo:**

analizar el concepto de bienestar socioemocional en el contexto del cuidado en salud.

**Método:**

estudio teórico de análisis de concepto guiado por el método de Walker y Avant, organizado en ocho etapas y operativizado mediante una revisión de alcance. La búsqueda se realizó en siete fuentes de datos, lo que dio como resultado la selección de siete estudios para conformar el corpus literario.

**Resultados:**

el bienestar socioemocional está caracterizado por atributos esenciales como el equilibrio emocional, la percepción de apoyo social, la sensación de controlar la propia vida y habilidades de regulación emocional. Los antecedentes incluyeron resiliencia, apoyo social, autoconocimiento, entorno favorable, estabilidad emocional, autonomía y toma de decisiones, así como vínculos interpersonales positivos. Como resultado, se observaron mejoras en la calidad de vida, un mayor compromiso social, una reducción de los síntomas psicosociales y un mejor desempeño en las actividades cotidianas.

**Conclusión:**

el estudio mostró que el bienestar socioemocional es un concepto multifacético que trasciende la salud mental individual y abarca dimensiones sociales, emocionales y culturales. Su integración en la atención sanitaria requiere enfoques interdisciplinarios que consideren los factores individuales y sociales, promoviendo una atención holística y centrada en el paciente.

## Destacados

**(1)** El bienestar socioemocional es multifacético y multidimensional. **(2)** El bienestar socioemocional contribuye a una mejor calidad de vida. **(3)** Tener equilibrio emocional es esencial para gestionar las emociones de forma adaptativa. **(4)** El apoyo social contribuye directamente a la resiliencia emocional. **(5)** Medir el bienestar socioemocional implica desafíos tanto conceptuales como metodológicos.

## Introducción

El bienestar ha cobrado una relevancia cada vez mayor en el campo de la salud, especialmente a medida que la atención al cuidado se expande más allá de los aspectos puramente biológicos y comienza a abarcar dimensiones emocionales y sociales^1^. Esta perspectiva holística favorece prácticas más humanas y centradas en la persona, con un impacto directo en la calidad del cuidado prestado.

Comprender el bienestar implica reconocer que la salud mental no se limita a la ausencia de trastornos, sino que abarca el equilibrio emocional, el bienestar psicológico y la capacidad de afrontar los desafíos de la vida cotidiana. Por lo tanto, está intrínsecamente relacionado con la capacidad de cada individuo para afrontar el estrés, trabajar de forma productiva y contribuir a su comunidad^2^ ^-^ ^3^.

La Organización Mundial de la Salud (OMS)^4^ ^)^ define la salud mental como “un estado de bienestar en el que el individuo desarrolla sus capacidades, afronta las tensiones normales de la vida, trabaja de forma productiva y contribuye a su comunidad”. Este concepto abarca dimensiones emocionales, psicológicas y sociales, e influye directamente en cómo las personas piensan, sienten, actúan, afrontan el estrés, construyen relaciones interpersonales y toman decisiones saludables, y estos componentes son esenciales e indisociables del bienestar^3^.

A lo largo del tiempo, el concepto de bienestar ha evolucionado desde una visión restringida a la ausencia de enfermedad hacia enfoques más complejos, como el bienestar subjetivo y el bienestar psicológico, que consideran, respectivamente, la satisfacción personal y la plena realización de las potencialidades humanas^3^. Esta evolución demuestra que tanto la vivencia de emociones positivas como la búsqueda de un propósito y crecimiento son esenciales para una vida plena. Sin embargo, su complejidad va más allá de estas dimensiones individuales e incluye también aspectos sociales y relacionales^5^.

Dentro de este contexto más amplio, el concepto de bienestar socioemocional ha surgido como una perspectiva integradora, que enfatiza la interacción entre los factores sociales y emocionales. Este constructo abarca la capacidad de afrontar los desafíos diarios, desarrollar resiliencia y mantener relaciones saludables^6^. Esta perspectiva refleja una comprensión más profunda y multifacética de este fenómeno, al concebir el bienestar socioemocional como un equilibrio entre la regulación emocional, el establecimiento de relaciones sociales y un sentido de propósito en la vida^7^. Estos enfoques refuerzan la comprensión del bienestar socioemocional como constructo multidimensional, esencial para el desarrollo integral y la calidad de vida (CdV) de los individuos.

A pesar del creciente uso del concepto de bienestar socioemocional en la literatura, aún no existe una definición consensuada, lo que limita su aplicación teórica y práctica. La complejidad de este constructo exige que investigadores y profesionales de la salud tengan en cuenta las experiencias subjetivas, las influencias culturales, sociales y las dinámicas familiares al desarrollar estrategias de promoción y prevención en materia de salud.

Además, la variedad de terminologías empleadas en la literatura pone de relieve la necesidad de una definición uniforme, que es fundamental para orientar tanto la práctica clínica como la investigación. En este contexto, se vuelve esencial aclarar el concepto de bienestar socioemocional, lo que subyace al objetivo de este estudio: analizar el concepto de bienestar socioemocional en el contexto de cuidado en salud.

## Método

### Tipo de estudio

Estudio teórico de análisis de concepto, desarrollado con base en el modelo propuesto por Walker y Avant^8^ y operativizado mediante revisión de alcance^9^. El análisis de concepto desarrollado es un enfoque sistemático ampliamente utilizado en la investigación de enfermería para aclarar conceptos complejos y ampliar su aplicación en la práctica clínica^10^.

El modelo^8^ ^)^ consta de ocho etapas: 1) selección del concepto, que consiste en elegir el fenómeno que estudiar; 2) establecimiento de los objetivos, cuando se definen los propósitos del análisis; 3) identificación de los posibles usos, que explora cómo se emplea el concepto en la literatura; 4) definición de los atributos esenciales, que establece las principales características del concepto; 5) identificación de un caso modelo, que ejemplifica el concepto de manera clara y representativa; 6) análisis de otros casos, incluidos casos limítrofes, relacionados y contrastantes, para resaltar diferencias y similitudes; 7) identificación de antecedentes y consecuentes, los factores que preceden y resultan del concepto; y 8) definición de referentes empíricos, que son los indicadores utilizados para medir el concepto en la práctica.

Para sustentar este análisis, se incorporó una revisión de alcance^9^ ^)^ (paso 3), registrada en *Open Science Framework* (https://osf.io/h5zr6/) bajo el DOI 10.17605/OSF.IO/H5ZR6, que contribuyó a las etapas posteriores del modelo.

### Recopilación de datos

Para la formulación de la pregunta de investigación, se utilizó la nemotecnia PCC: Población: No especificada; Concepto: Bienestar socioemocional; Contexto: Cuidados de salud. Con base en estas definiciones, se estableció la siguiente pregunta rectora: ¿Cómo se aborda el concepto de bienestar socioemocional en el contexto de los cuidados de salud? Las búsquedas se llevaron a cabo en noviembre y diciembre del 2024, con la estrategia principal desarrollada en conjunto con un profesional bibliotecario y ajustada para las ocho fuentes de datos PubMed, EMBASE (*Excerpta Medica dataBASE*), *Web of Science*, *Cumulative Index to Nursing and Allied Health Literature* (CINAHL), MEDLINE (*Medical Literature Analysis and Retrieval System Online*), Scopus (Elsevier), Biblioteca Virtual en Salud (BVS) y *Google* ^*®*^ Académico, combinado con los operadores booleanos AND y OR (Figura 1). 

**Figura 1:** Estrategias de búsqueda. Curitiba, PR, Brasil, 2025

Fuente de datos	Estrategias de búsqueda
BVS*	(“Bem-estar socioemocional” OR “Bienestar socioemocional” OR “Socioemotional well-being” OR “Bem-estar” OR “Bienestar” OR “Well-being”) AND (“Cuidados de saúde” OR “Cuidados de salud” OR “Atenção à saúde” OR “Atención a la salud”)
EMBASE^†^	(‘Socioemotional well-being’:ti,ab,kw OR ‘Well-being’:ti,ab,kw)) AND (‘Health care’:ti,ab,kw OR ‘Health care’/exp OR ‘Healthcare’:ti,ab,kw OR ‘Health services’:ti,ab,kw OR ‘Health services’/exp OR ‘Delivery of health care’:ti,ab,kw OR ‘Delivery of health care’/exp)
MEDLINE^‡^	(‘Socioemotional well-being’:ti,ab,kw OR ‘Well-being’:ti,ab,kw)) AND (‘Health care’:ti,ab,kw OR ‘Health care’/exp OR ‘Healthcare’:ti,ab,kw OR ‘Health services’:ti,ab,kw OR ‘Health services’/exp OR ‘Delivery of health care’:ti,ab,kw OR ‘Delivery of health care’/exp)
PubMed	(“Socioemotional well-being”[Title/Abstract] OR “Well-being”[Title/Abstract]) AND (“Health care”[MeSH Terms] OR “Health care”[Title/Abstract] OR “Healthcare”[Title/Abstract] OR “Health services”[MeSH Terms] OR “Health services”[Title/Abstract] OR “Delivery of health care”[MeSH Terms] OR “Delivery of health care”[Title/Abstract])
Scopus (Elsevier)	TITLE-ABS-KEY((“Socioemotional well-being” OR “Well-being”) AND TITLE-ABS-KEY (“Health care” OR “Healthcare” OR “Health services” OR “Delivery of health care”))
*Web of Science*	ALL=(“Socioemotional well-being” OR “Well-being”) AND ALL=(“Health care” OR “Healthcare” OR “Health services” OR “Delivery of health care”)
CINAHL^§^	(“Bem-estar socioemocional” OR “Bienestar socioemocional” OR “Socioemotional well-being” OR “Bem-estar” OR “Bienestar” OR “Well-being”) AND (“Cuidados de saúde” OR “Cuidados de salud” OR “Atenção à saúde” OR “Atención a la salud”)
*Google* ^*®*^ **Académico**	(“Bem-estar socioemocional” OR “Bienestar socioemocional” OR “Socioemotional well-being” OR “Bem-estar” OR “Bienestar” OR “Well-being”) AND (“Cuidados de saúde” OR “Cuidados de salud” OR “Atenção à saúde” OR “Atención a la salud”)

*BVS = Biblioteca Virtual en Salud; ^†^EMBASE = *Excerpta Medica dataBASE*; ^‡^MEDLINE = *Medical Literature Analysis and Retrieval System Online*; ^§^CINAHL = *Cumulative Index to Nursing and Allied Health Literature*

### Criterios de selección

Se delimitaron los siguientes criterios de inclusión: estudios accesibles en su totalidad, publicados entre el 2014 y el 2024, un período definido para abarcar producciones recientes, en portugués, español e inglés; que contuvieran la expresión “bienestar socioemocional” en el título, resumen o palabras clave para asegurar la relevancia de los artículos para el tema central de la investigación. Se excluyeron los siguientes estudios: cartas al editor, editoriales, resúmenes, encuestas de opinión y duplicados.

Todos los artículos identificados se exportaron a la plataforma de gestión de revisiones *Rayyan* ^*®*^ . Dos revisores evaluaron de forma ciega los títulos, resúmenes y descriptores basándose en los criterios de elegibilidad. Las discrepancias fueron resueltas por un tercer revisor. Además de la selección en las bases de datos, se realizó una búsqueda manual en las listas de referencias de los estudios incluidos. 

Selección de los estudios, procesamiento y análisis de datos

La búsqueda identificó 13.728 estudios, de los cuales 1.883 eran duplicados. Después de revisar títulos, resúmenes y palabras clave relacionados con el término bienestar socioemocional, se excluyeron 11.785 estudios. Restaron 60 estudios para su lectura completa, de los cuales 53 se descartaron por no cumplir los criterios de inclusión. Solo se consideraron los artículos que abordaban explícitamente el concepto de bienestar socioemocional, lo que dio como resultado siete estudios incluidos en la revisión final.

Tras la selección, los estudios se organizaron de la siguiente manera: autores y año, país, población y muestra, objetivo principal, concepto de bienestar socioemocional en el contexto de la salud y dimensiones observadas, como se ilustra en la Figura 2. La definición de los atributos esenciales del bienestar socioemocional se logró mediante la siguiente pregunta: ¿Cuáles son las características fundamentales que definen el bienestar socioemocional? Para identificar sus antecedentes, se formuló la siguiente pregunta: ¿Qué eventos preceden al bienestar socioemocional? Finalmente, para determinar sus consecuentes, se consideró la siguiente pregunta: ¿Qué eventos resultan del bienestar socioemocional?

**Figure 2:** Characterization of selected studies. Curitiba, PR, Brazil, 2025

Autores y año	País	Población y muestra	Objetivo principal	Concepto de bienestar socioemocional en salud	Dimensiones observadas
Ashton, et al., 2024^11^	Nueva Zelanda	Padres (n=257) e hijos (n=285)	Evaluar el impacto a largo plazo (12 meses) del programa comunitario	Efecto positivo de actividades físicas comunitarias en bienestar socioemocional	Lazos familiares; reducción del estrés; apoyo emocional; ambiente positivo
Tunalilar, et al., 2024^12^	EE. UU.*	Proveedores (2,027) de hogares de acogida para adultos	Monitorear cambios en el involucramiento familiar tras la pandemia	Efecto de la reducción del involucramiento familiar en bienestar socioemocional	Dependencia del involucramiento; soledad; estrés; importancia de lazos afectivos
Us, et al., 2024^1^	Turquía	Hijos (n=600) de profesionales de la salud	Explorar problemas socioemocionales y conductuales de niños	Impacto emocional derivado del estrés familiar y social	Vulnerabilidad infantil; impacto del estrés; influencia del trabajo materno
Timmer, et al., 2022^13^	Australia, Suiza, EE. UU.* y Reino Unido	Revisión	Recomendaciones para incluir bienestar socioemocional en rehabilitación audiológica	Impacto de pérdida auditiva en autoestima, exclusión social y salud mental	Autoestima reducida; exclusión social; ansiedad; importancia del apoyo emocional
Siette, et al., 2021^14^	Australia	Adultos mayores (n=21) con atención domiciliaria	Impacto de la pandemia en calidad de vida y redes sociales	Bienestar socioemocional afectado por aislamiento social	Aislamiento; soledad; depresión; importancia de redes de apoyo
Fenwick, et al., 2020^15^	Singapur y Australia	Adultos (n=220) con retinopatía diabética	Validar índice de utilidad para retinopatía diabética	Impacto de pérdida de visión en bienestar socioemocional	Autoestima; relaciones sociales; percepción de pérdidas; impacto de enfermedad crónica
Pruner, et al., 2020^16^	EE. UU.*	Padres adoptivos/biológicos (n=25) de niños con riesgo de TEAF	Identificar características de intervención temprana favorables y desafiantes	Bienestar socioemocional de padres; reducción de estrés y ansiedad	Equilibrio emocional; dinámica familiar; necesidad de apoyo; relación padres-hijos

*USA = United States of America, †UK = United Kingdom; ‡FASD = Fetal Alcohol Spectrum Disorders

### Uso de inteligencia artificial

Los autores reconocen haber utilizado ChatGPT (https://chatgpt.com) para elaborar la Figura 5, que presenta un diagrama conceptual que ilustra las relaciones entre los factores asociados con el bienestar socioemocional. Los autores han editado los datos y la figura para perfeccionarlos y mejorarlos.

## Resultados

La presentación de los resultados está de acuerdo con los pasos de análisis de concepto según Walker y Avant^8^.

### Selección y objetivo

El “bienestar” se define a menudo como un estado de equilibrio físico, mental y social en el que el individuo se siente sano, cómodo y en armonía consigo mismo y con su entorno. Este estado implica aspectos emocionales y psicológicos, como la felicidad, la seguridad y la realización, así como componentes físicos, como la salud y la vitalidad^5^.

Además, el bienestar puede extenderse al contexto social, abarcando relaciones interpersonales saludables y un sentido de pertenencia a la comunidad. En general, representa el estado de bienestar de manera holística, incluida la ausencia de sufrimiento, una buena CdV y la capacidad de afrontar los desafíos de manera adaptativa y positiva^5^.

Aunque la OMS no proporciona una definición específica de “bienestar socioemocional”, el concepto parece estar estrechamente relacionado con la salud mental, una vez que implica la capacidad de gestionar las emociones, establecer relaciones interpersonales saludables y mantener el equilibrio emocional. Según el *Australian Institute of Health and Welfare* ^17^, el bienestar socioemocional se describe como una parte esencial del bienestar mental y social, que va más allá de la ausencia de enfermedad e incluye la capacidad de mantener relaciones positivas, gestionar las emociones y participar activamente en la comunidad, abarcando la resiliencia emocional, el sentido de pertenencia y el apoyo social.

La falta de consenso en las definiciones del bienestar socioemocional pone de relieve su complejidad y refuerza la necesidad de una conceptualización clara y contextualizada. Esta falta de consenso limita su aplicación en investigaciones e intervenciones, lo que dificulta las comparaciones y generalizaciones. Establecer una definición coherente, flexible y aplicable a diferentes contextos es esencial para consolidar el concepto, orientar prácticas de cuidado eficaces y promover el bienestar socioemocional de forma amplia y significativa.

### Identificación de los posibles usos del concepto

Este tema identifica los posibles usos del concepto de bienestar, basándose en los siete estudios seleccionados en la tercera etapa, que exploran sus aplicaciones, características e implicaciones prácticas en diferentes contextos.

Un estudio realizado^1^ ^)^ en Turquía utilizó el concepto de bienestar socioemocional para investigar cómo el estrés y la sobrecarga de trabajo de madres profesionales de la salud, durante la pandemia de COVID-19, afectaron el equilibrio emocional y conductual de sus hijos. El estudio resaltó que el bienestar socioemocional de los niños se veía comprometido, lo que se traducía en mayores índices de ansiedad, depresión y problemas de conducta. De este modo, se aplicó el bienestar socioemocional para comprender cómo los factores externos, como el estrés parental, afectan la salud mental de los niños y refuerzan la necesidad de políticas de apoyo a las familias en situaciones de crisis.

En un contexto diferente, un estudio^15^ realizado en Singapur y Australia abordó indirectamente el bienestar socioemocional al validar un índice de utilidad para pacientes con retinopatía diabética. Aunque el foco principal era la CdV relacionada con la salud, el estudio destacó que la pérdida de visión puede conducir al aislamiento social y a la depresión, afectando el bienestar socioemocional de los pacientes. En este caso, el concepto se utilizó para enfatizar la importancia de considerar los aspectos emocionales y sociales en el tratamiento de las afecciones crónicas.

En los Estados Unidos de América (EE. UU.), investigadores^16^ ^)^ exploraron las experiencias de padres de niños con trastorno del espectro alcohólico fetal (TEAF), utilizando el concepto de bienestar socioemocional para entender cómo el estrés y la sobrecarga emocional afectan la salud mental de los padres. El estudio reveló que muchos padres afrontan sentimientos de culpa, estrés y aislamiento, lo que compromete su equilibrio emocional. Aquí, el concepto se aplicó para resaltar la necesidad de apoyo psicológico y práctico para los padres, para promover un ambiente familiar más saludable.

En Australia^14^ se investigó el impacto de la pandemia de COVID-19 en la CdV de adultos mayores que reciben cuidados comunitarios, utilizando el concepto de bienestar socioemocional para explorar cómo el aislamiento social y la interrupción de los servicios afectaron la salud mental de los adultos mayores. El estudio mostró que muchas personas mayores reportaron soledad y depresión, lo que destaca la importancia de las estrategias para mantener el contacto social y el apoyo emocional. En este contexto, el concepto se aplicó para enfatizar la necesidad de enfoques integrados que consideren tanto el cuidado físico como el emocional.

Otro estudio^13^, una colaboración entre Australia, Suiza, Estados Unidos y Reino Unido se centró en el bienestar socioemocional de adultos con pérdida auditiva. El concepto se utilizó para analizar cómo la pérdida auditiva puede provocar aislamiento social y depresión, afectando la CdV. El estudio recomendó incluir estrategias para promover el bienestar socioemocional, como el asesoramiento psicológico y el apoyo social, en los programas de rehabilitación audiológica. El concepto se aplicó para resaltar la importancia de un enfoque holístico en el tratamiento de las afecciones crónicas.

En los EE. UU.^12^, se analizó el involucramiento familiar entre los residentes adultos de hogares de acogida, utilizando el concepto de bienestar socioemocional para explorar cómo la reducción del contacto familiar durante la pandemia afectó la salud mental de los residentes. El estudio mostró que muchos residentes manifestaron sentimientos de abandono y soledad, lo que resalta la importancia del involucramiento familiar para el equilibrio emocional. En este caso, el concepto se aplicó para reforzar la necesidad de políticas que fortalezcan los lazos familiares y promuevan el bienestar socioemocional.

En Nueva Zelanda^11^ se evaluaron los resultados de un programa comunitario de actividad física entre padres e hijas, utilizando el concepto de bienestar socioemocional para explorar cómo la interacción social y la actividad física afectan la salud mental y emocional de los participantes. El estudio demostró que el programa promovió mejoras significativas en el bienestar socioemocional, fortaleciendo los lazos familiares y reduciendo el estrés. El concepto se aplicó para resaltar la importancia de los programas que integran la actividad física y la interacción social para promover la salud mental.

Al analizar estos estudios, se refuerza la idea de que el bienestar socioemocional es un concepto multidimensional, esencial para la CdV en diferentes momentos y contextos. Desde el impacto de la pandemia en la salud mental infantil hasta los desafíos que enfrentan los adultos mayores y las personas con enfermedades crónicas, el equilibrio emocional y social emerge como un factor crucial para el desarrollo humano.

### Atributos esenciales

Definir los atributos esenciales es un paso fundamental en el análisis de concepto, una vez que son características que aclaran la esencia del concepto, permitiendo reconocerlo, diferenciarlo y validar su aplicabilidad en diferentes contextos^8^. El análisis de los estudios seleccionados reveló los atributos esenciales del bienestar socioemocional.

*Equilibrio emocional*

El equilibrio emocional se refiere a la capacidad de gestionar las emociones de forma estable y adaptativa, minimizando los impactos negativos de la ansiedad, del estrés y de la tristeza. Es esencial para mantener la salud mental e influye directamente en cómo las personas afrontan los desafíos cotidianos^14^ ^,^ ^16^.

La importancia del equilibrio emocional se hace evidente en diferentes contextos. Un estudio^14^ que monitoreó a adultos mayores en cuidados comunitarios durante la pandemia utilizó escalas de salud mental y entrevistas semiestructuradas para analizar la relación entre el equilibrio emocional y la CdV. Los resultados indicaron que los adultos mayores con mayor equilibrio emocional presentaban niveles más bajos de soledad y depresión, lo que demuestra la relevancia de este atributo para el bienestar socioemocional, especialmente en situaciones de vulnerabilidad.

Más allá del impacto individual, el equilibrio emocional también influye en el ambiente familiar. Un estudio^16^ sobre padres de niños con o en riesgo de padecer TEAF identificó que el equilibrio emocional de los padres afecta directamente el bienestar de las familias. Los padres con mayor control emocional mostraron una mejor adaptación a las dificultades que enfrentaban, lo que contribuye a la salud emocional de todos los miembros de la familia. Estos hallazgos refuerzan la idea de que el equilibrio emocional fortalece al individuo y desempeña un papel crucial en las relaciones interpersonales y en la dinámica familiar. 

*Percepción del apoyo social*

La percepción del apoyo social se relaciona con la sensación de apoyo recibida de familiares, amigos y comunidad. Desempeña un papel central en la resiliencia emocional y en el afrontamiento de las dificultades y promueve un sentimiento de pertenencia y seguridad. Además, sentirse apoyado está directamente asociado a la confianza y a la estabilidad emocional^11^ ^,^ ^13^.

La percepción del apoyo social es un factor determinante para el bienestar emocional, especialmente en los grupos vulnerables. En pacientes con pérdida auditiva, sentirse apoyado redujo los niveles de estrés y facilitó la adaptación a las limitaciones, como lo demuestra una investigación que utilizó cuestionarios y pruebas psicológicas^13^. En el contexto familiar, el apoyo fue esencial para el bienestar socioemocional de residentes en hogares de acogida, mientras que su ausencia, especialmente durante la pandemia de COVID-19, se asoció a mayor estrés y dificultades emocionales^12^.

Además del entorno familiar, el apoyo de la comunidad también desempeña un papel fundamental. Un estudio que analizó la participación de padres e hijos en un programa comunitario de actividad física demostró que el apoyo social fortaleció los lazos familiares y promovió un sentido de pertenencia, contribuyendo al bienestar socioemocional de los participantes^11^.

*Sensación de control sobre la propia vida*

La sensación de control sobre la propia vida está relacionada con la percepción de autonomía y la capacidad de tomar decisiones que influyen en el propio bienestar. Este atributo está fuertemente asociado a la autoconfianza, la independencia y la satisfacción personal^15^.

La percepción de control sobre la propia vida demuestra un impacto significativo en la adaptación a condiciones adversas. Un estudio^15^ con pacientes con retinopatía diabética, realizado mediante experimentos de elección discreta y relatos de CdV, observó que aquellos que se sentían en control de sus propias decisiones de salud relataron una mayor satisfacción y una adaptación más positiva a la condición crónica. Esto pone de relieve la importancia de sentirse en control para el bienestar emocional y la resiliencia frente a afecciones de salud complicadas.

En un contexto similar, una investigación^14^ realizada con adultos mayores en cuidados comunitarios durante la pandemia de COVID-19 destacó la relevancia de la autonomía en la salud emocional. Quienes mantuvieron una percepción de control sobre sus vidas presentaron menos ansiedad y una mejor CdV, lo que sugiere que la capacidad de gestionar la propia vida, especialmente en momentos de gran incertidumbre, es fundamental para mantener la salud emocional.

*Habilidades de regulación emocional*

Las habilidades de regulación emocional implican la capacidad de reconocer, comprender y gestionar las emociones de forma adaptativa, lo que permite a las personas enfrentar desafíos sin desarrollar respuestas de estrés o ansiedad desproporcionadas^13^.

La relevancia de este atributo se ha demostrado en diferentes poblaciones. Una investigación^16^ con padres de niños con TEAF, basada en entrevistas y escalas de regulación emocional, señaló que aquellos con mayor dominio de estas habilidades presentaron una mejor adaptación a desafíos complejos, lo que favorece el equilibrio psicológico y la resiliencia.

Además, una revisión^13^ sobre el impacto de la pérdida auditiva en el bienestar socioemocional de adultos destacó que la regulación emocional desempeña un papel esencial en la adaptación a la pérdida auditiva. Los pacientes que demostraron una mayor capacidad para gestionar sus emociones afrontaron los desafíos de la afección de una manera más positiva, lo que demuestra que el desarrollo de estas habilidades puede mitigar los impactos negativos de las limitaciones físicas en la CdV. La Figura 3 muestra los atributos esenciales enumerados.

**Figura 3:** Atributos esenciales del bienestar socioemocional. Curitiba, PR, Brasil, 2025

Atributos	Descripción	Estudios relacionados
Equilibrio emocional	Capacidad para gestionar las emociones	Siette, et al. (2021): Adultos mayores que mantuvieron el equilibrio emocional relataron menos soledad y depresión^14^.
Percepción del apoyo social	Sensación de pertenencia y conexión	Timmer, et al. (2022): Pacientes con pérdida auditiva que recibían apoyo social presentaron mejor bienestar emocional^13^.
Sensación de control sobre la propia vida	Autonomía y capacidad de tomar decisiones	Fenwick, et al. (2020): Pacientes con percepción de control de su propia condición relataron una mayor calidad de vida^15^.
Habilidades de regulación emocional	Capacidad para identificar y regular las emociones	Pruner, et al. (2020): Padres que desarrollaron habilidades de regulación emocional afrontaron mejor el estrés^16^.

Estos atributos son fundamentales para el bienestar socioemocional y se manifiestan en diferentes contextos. La percepción de apoyo social fortalece el sentido de pertenencia y conexión, mientras que la sensación de control sobre la propia vida promueve la autonomía y reduce la ansiedad. El equilibrio emocional permite afrontar los estresores de forma adaptativa, repercutiendo positivamente en la salud mental. Además, las habilidades de regulación emocional capacitan a los individuos para gestionar sus emociones de manera efectiva, previniendo reacciones desproporcionadas y promoviendo el bienestar general.

### Caso modelo

Los casos son ejemplos utilizados para ilustrar y aclarar el concepto en estudio. Ayudan a diferenciar el concepto de los términos relacionados y a mostrar cómo se puede aplicar en diferentes contextos^8^.

El caso modelo representa la manifestación completa del concepto de bienestar socioemocional. L.P.M., 45 años, profesora universitaria, a quien recientemente se le diagnosticó cáncer de mama en etapa temprana, ha estado experimentando ataques de ansiedad desde su diagnóstico, los cuales se intensifican por la noche. Comenzó un seguimiento profesional con psicoterapia, práctica regular de meditación guiada y participación en un grupo de artes de la comunidad. A lo largo de ocho meses, desarrolló atributos esenciales de bienestar socioemocional. L.P.M. demuestra capacidad para afrontar situaciones complejas asociadas al tratamiento, mantiene lazos significativos con familiares y colegas, se siente apoyada por su red social y expresa sentimientos de esperanza en relación con el futuro. Además, demuestra una regulación emocional saludable en situaciones estresoras, es capaz de resignificar su experiencia de enfermedad y mantiene un nivel satisfactorio de participación en actividades diarias. Como resultado, se observa una reducción de la angustia emocional, un mejor desempeño en las actividades cotidianas y una mayor CdV. Este caso refleja la plena manifestación del concepto de bienestar socioemocional.

### Análisis de otros casos

*Caso limítrofe*

El caso limítrofe contiene algunos, pero no todos los atributos esenciales del concepto^8^. L.G.P., de 42 años, diagnosticada con depresión provocada por estresores laborales, comenzó un seguimiento con terapia cognitivo-conductual y tratamiento medicamentoso. Además, comenzó a realizar actividades físicas con regularidad y a disfrutar de actividades de ocio. A pesar de la mejoría inicial, con una actitud de afrontamiento más positiva y una mayor sensación de control sobre su propia vida, aún presenta inestabilidad emocional (estado de ánimo depresivo, irritabilidad, ansiedad) y dificultades en la regulación emocional, especialmente ante las demandas relacionadas con el trabajo. Aunque los síntomas depresivos han disminuido, persisten fluctuaciones psicológicas significativas. Se trata, por lo tanto, de un caso limítrofe, en el que están presentes algunos atributos del bienestar socioemocional, pero no de forma integral. 

*Caso contrario*

El caso contrario ejemplifica el “no concepto”, es decir, la ausencia total o casi total de los atributos esenciales del concepto^8^. C.O.R., de 38 años, que recientemente se mudó del interior a São Paulo-SP, presenta trastornos de ansiedad y depresión. Aunque consultó con un psiquiatra, no siguió las recomendaciones médicas ni buscó seguimiento psicológico o terapéutico adicional. Presenta episodios recurrentes de irritabilidad, estado de ánimo depresivo, ansiedad y sensación de pérdida de control. Vive aislado y sin redes de apoyo, y pasa gran parte del tiempo confinado en su habitación, sin participar en actividades sociales o de ocio. En este contexto, los síntomas de ansiedad y depresión se ven agravados por la ausencia de apoyo social y de estrategias de afrontamiento adecuadas. Esto pone de manifiesto una baja CdV, con ausencia de los atributos esenciales del bienestar socioemocional.

*Caso relacionado*

El caso relacionado ilustra una situación que está asociada al concepto de bienestar socioemocional^8^. G.T., de 28 años, estudiante del sexto curso de medicina, presenta estrés crónico, insomnio e inestabilidad emocional durante gran parte del año académico. Cree que padece ansiedad y depresión. Sin embargo, al final de la temporada de pruebas, se observa una mejora significativa del cuadro, debido a la reducción del estrés y a la reanudación de las actividades físicas, las prácticas de ocio y la interacción social con los amigos. Este caso está relacionado con el concepto, pero no abarca plenamente sus atributos esenciales, una vez que no se busca una evaluación profesional ni un seguimiento terapéutico, que son esenciales para el desarrollo completo del bienestar socioemocional.

### Antecedentes y consecuentes

Los antecedentes se refieren a las causas o circunstancias que preceden a la promoción del bienestar socioemocional^8^. El análisis de los estudios reveló que varios factores contribuyen al desarrollo de este concepto.

*Resiliencia*

La capacidad de superar adversidades y adaptarse a circunstancias desafiadoras desempeña un papel esencial en el equilibrio emocional y en el mantenimiento del bienestar socioemocional^15^. Los individuos más resilientes tienden a tener mayor control sobre su propia vida y desarrollan estrategias más efectivas para afrontar situaciones estresantes, lo que favorece su adaptación a diferentes contextos y contribuye a una mejor CdV^15^.

*Apoyo social*

El apoyo emocional y práctico que ofrecen la familia, los amigos o las redes comunitarias tiene un impacto directo en la reducción del estrés y en el fortalecimiento del bienestar. La presencia de una red de apoyo contribuye a afrontar los desafíos y promueve un sentido de pertenencia, lo que favorece la estabilidad emocional^14^. Además, los entornos que fomentan la interacción social fortalecen los lazos interpersonales y crean condiciones propicias para un desarrollo socioemocional saludable^13^.

*Autoconocimiento*

Las personas con un mayor grado de autoconocimiento afrontan mejor los desafíos diarios, evitando respuestas impulsivas o desproporcionadas. Este factor está directamente relacionado con el desarrollo de la resiliencia y el fortalecimiento de las habilidades emocionales, lo que contribuye a una mejor adaptación a diferentes circunstancias de la vida^16^.

*Entorno favorable*

Un entorno que ofrece seguridad emocional, apoyo social y acceso a recursos adecuados influye directamente en el bienestar socioemocional. Los entornos acogedores promueven una mayor estabilidad emocional y facilitan la creación de relaciones interpersonales positivas. La presencia de espacios que fomentan interacciones saludables y brindan apoyo continuo ayuda a fortalecer la regulación emocional y la capacidad de afrontar los desafíos^16^.

*Estabilidad emocional*

Mantener un estado emocional equilibrado ante situaciones adversas es un factor esencial para el bienestar socioemocional. Los individuos emocionalmente estables presentan un mayor control sobre sus acciones y reacciones, lo que favorece una adaptación más positiva a diferentes circunstancias^15^. Esta estabilidad está relacionada con la capacidad de regular las emociones y afrontar los desafíos de manera racional y resiliente.

*Autonomía y toma de decisiones*

La percepción de control sobre la propia vida está directamente asociada al bienestar socioemocional. Tener autonomía para tomar decisiones, especialmente en la gestión de la propia salud y en la organización de la vida cotidiana, reduce los niveles de ansiedad y promueve una mayor satisfacción personal. Sentirse en control de las propias decisiones fortalece la autoconfianza y proporciona una mayor sensación de logro y propósito^15^.

*Relaciones interpersonales positivas*

Las relaciones saludables y las interacciones sociales significativas son fundamentales para el bienestar socioemocional. Los lazos interpersonales basados en la confianza y en el apoyo mutuo ofrecen seguridad emocional y contribuyen a construir un sentido de pertenencia^14^. Los entornos que animan estas conexiones fortalecen la percepción de apoyo social y promueven una mayor estabilidad emocional a lo largo del tiempo.

Los consecuentes del bienestar socioemocional se refieren a los resultados que surgen como consecuencia de este estado^8^. 

*Mejora en la calidad de vida*

El bienestar socioemocional contribuye a un estado general más equilibrado, lo que se refleja directamente en la CdV. Los individuos que presentan mayor estabilidad emocional y un apoyo social adecuado afrontan desafíos con más resiliencia, demuestran mayor satisfacción personal y mantienen una perspectiva más positiva de la vida. Esto se percibe en la adaptación a condiciones de salud crónicas, en el afrontamiento de dificultades familiares y en la gestión del estrés cotidiano^14^.

*Mayor participación social*

Las personas que experimentan un estado emocional equilibrado tienden a buscar y mantener conexiones sociales de manera más activa, participan en actividades comunitarias, establecen lazos más sólidos con familiares y amigos, y desarrollan un sentido de pertenencia. Esta interacción favorece el intercambio de apoyo y crea un círculo virtuoso en el que el contacto social refuerza el bienestar emocional^11^.

*Reducción de síntomas psicosociales*

La capacidad de regular emociones de manera efectiva y la presencia de una red de apoyo confiable permiten a los individuos afrontar mejor las situaciones adversas, minimizando los impactos negativos en la salud mental. En grupos vulnerables, como los de adultos mayores, enfermos crónicos o individuos que afrontan cambios significativos en su vida, la presencia de bienestar socioemocional puede ser un factor determinante en la prevención de trastornos psicológicos^13^.

*Mejora del desempeño en las actividades diarias*

Los individuos con mayor equilibrio emocional demuestran más motivación, concentración y capacidad de adaptación, lo que se refleja en un mejor rendimiento en el entorno escolar, en el trabajo y en las interacciones familiares. Los niños y adultos con un elevado bienestar socioemocional presentan un mayor compromiso con sus tareas y un enfoque más positivo ante los desafíos, lo que favorece un funcionamiento más productivo y eficiente^1^.

Los consecuentes del bienestar socioemocional demuestran sus amplios beneficios, que abarcan aspectos emocionales, sociales y funcionales. La estabilidad psicológica favorece la participación activa en la vida social y el mantenimiento de una buena CdV, lo que crea un círculo positivo en el que las personas emocionalmente equilibradas tienden a desarrollar relaciones más sanas y a afrontar desafíos con mayor confianza. Por lo tanto, promover y fortalecer el bienestar socioemocional se convierte en una estrategia esencial para mejorar la salud mental y el bienestar general de la población.

La Figura 4 presenta los antecedentes y consecuentes del bienestar socioemocional.

**Figura 4:** Antecedentes y consecuentes del bienestar socioemocional. Curitiba, PR, Brasil, 2025

Categoría	Elementos	Descripción	Estudios relacionados
Antecedentes	Resiliencia	Afrontar la adversidad, como el estrés parental durante la pandemia.	Us, et al. (2024): La resiliencia mitigó efectos del estrés en niños cuyas madres son profesionales de la salud^1^.
	Apoyo social	Las redes de apoyo promueven la estabilidad emocional.	Tunalilar, et al. (2024): El apoyo familiar es esencial para el bienestar en hogares adoptivos para adultos^12^.
	Autoconocimiento	La conciencia emocional ayuda a regular el estrés.	Pruner, et al. (2020): Padres de niños con trastorno del espectro alcohólico fetal destacaron la importancia del autoconocimiento^16^.
	Entorno favorable	Los entornos seguros y con abundantes recursos fortalecen el bienestar socioemocional.	Ashton, et al. (2024): Programas comunitarios promueven un entorno positivo^11^.
	Estabilidad emocional	La capacidad de mantener el equilibrio ante las emociones.	Fenwick, et al. (2020): La regulación emocional en situaciones adversas favorece la adaptación positiva en diferentes circunstancias^15^.
	Autonomía	El control sobre las decisiones aumenta la confianza y el sentido de propósito.	Fenwick, et al. (2020): La autonomía fortalece la autoestima y la satisfacción^15^.
	Relaciones interpersonales positivas	Relaciones saludables y de apoyo que contribuyen al bienestar socioemocional.	Siette, et al. (2021): Adultos mayores con apoyo e interacción social significativa presentan mayor estabilidad emocional a lo largo del tiempo^14^.
Consecuentes	Mejora en la calidad de vida	Mayor satisfacción y bienestar.	Siette, et al. (2021): Adultos mayores con apoyo presentan mejor calidad de vida^14^.
	Compromiso social	Mayor interacción y participación social.	Ashton, et al. (2024): Los programas de actividades promueven un mayor involucramiento^11^.
	Reducción de los síntomas psicológicos	Menos ansiedad y depresión.	Timmer, et al. (2022): Pacientes con apoyo relataron mejora emocional^13^.
	Mejor desempeño	Mayor productividad y funcionalidad.	Us, et al. (2024): Niños con mayor bienestar presentaron mejor rendimiento escolar^1^_._

La Figura 5 presenta un diagrama conceptual que ilustra las relaciones entre los factores asociados con el bienestar socioemocional, organizados en tres niveles: antecedentes, atributos y consecuentes.

**Figura 5:** Diagrama conceptual del bienestar socioemocional. Curitiba, PR, Brasil, 2025

Fuente: OpenAI

## Discusión

### Definición de referentes empíricos

Los referentes empíricos son las herramientas de medición que demuestran la ocurrencia del concepto, lo que requiere la identificación de indicadores observables, métodos de medición y estándares de manifestación^8^. 

Los estudios analizados aportan evidencias sobre cómo se manifiesta el bienestar socioemocional y cómo se ve influenciado por factores externos, lo que permite una comprensión más estructurada de sus características fundamentales. Sin embargo, su medición aún enfrenta desafíos conceptuales y metodológicos, ya que no existe ningún instrumento que capte integralmente la complejidad de las experiencias asociadas a este concepto.

La salud mental y el bienestar son constructos multideterminados, moldeados por factores biológicos, sociales y culturales que influyen en la percepción, la vivencia emocional y las estrategias de afrontamiento e interacción social de los individuos. Dado que cada contexto cultural define sus propios parámetros sobre lo que constituye la salud mental, la sensibilidad a estos matices es imperativa para la relevancia y la eficacia de cualquier intervención. Esta complejidad inherente exige el desarrollo de instrumentos de evaluación capaces de captar sus múltiples dimensiones para realizar una medición integral y adecuadamente contextualizada del concepto^2^.

Los estudios revisados utilizaron instrumentos estandarizados para evaluar diferentes dimensiones del bienestar socioemocional, incluida la CdV, el apoyo social y el bienestar emocional. Se utilizó la Escala de Redes Sociales de Lubben (LSNS-6) para medir el apoyo social percibido, centrándose en las redes de apoyo de familiares y amigos. A su vez, el EQ-5D-5L (EuroQoL-5D-5L) evaluó la calidad de vida relacionada con la salud (CVRS) en cinco dimensiones: movilidad, autocuidado, dolor/incomodidad, actividades habituales y síntomas de ansiedad/depresión^14^.

Se aplicaron otros instrumentos para medir aspectos específicos del bienestar socioemocional en diferentes poblaciones. La Escala Breve de Evaluación Social y Emocional (BITISEA), por ejemplo, se utilizó para evaluar la salud social y emocional de niños de hasta tres años de edad^1^. En el contexto de la atención auditiva, se han recomendado cuestionarios como el *Patient Health Questionnaire*-4 (PHQ-4), el Cuestionario de Aceptación y Acción ante la Pérdida Auditiva, el Cuestionario de Restricciones de Participación Social (SPARQ) y su versión reducida de cinco ítems (SIM), así como el Inventario de *Handicap* Auditivo para Adultos Mayores (HHIE-S), para investigar el impacto de la pérdida auditiva en el bienestar socioemocional^13^.

Además, un estudio multicéntrico^15^ incorporó el bienestar socioemocional en el desarrollo del *Disease-Related Utility Index* (DRU-I), una escala encaminada a evaluar la CdV relacionada con la audición. En esta escala, el bienestar socioemocional se consideró una de las cinco dimensiones centrales de la CdV de los pacientes. Sin embargo, la complejidad de este constructo requiere un enfoque más profundo que considere sus múltiples dimensiones y los factores que influyen en él, lo que permite una evaluación más integral y precisa.

Cabe resaltar que ninguno de los estudios revisados aplicó o desarrolló una escala que mida objetivamente el bienestar socioemocional. Las herramientas utilizadas evalúan aspectos correlacionados, como los síntomas de depresión, ansiedad y satisfacción hacia la vida, pero no captan el constructo de manera integral y específica. Esto pone de manifiesto una laguna en la literatura respecto a la medición directa del bienestar socioemocional.

El análisis de los estudios revisados revela que el bienestar socioemocional es un constructo multidimensional, influenciado por varios factores, como la adaptación al estrés^12^, la calidad de las relaciones interpersonales^15^ y el apoyo social^14^. Además, conceptos como resiliencia emocional^16^, autoestima^13^, apoyo familiar^11^ y adaptación social^1^ son fundamentales para promover el bienestar socioemocional, especialmente en contextos adversos, como las crisis de salud pública y las enfermedades crónicas. Estos factores tienen un impacto significativo en la salud mental de individuos de diferentes rangos de edad y son esenciales para desarrollar intervenciones más efectivas y personalizadas. 

Por lo tanto, es crucial que las estrategias para promover el bienestar socioemocional se integren en las prácticas de cuidados de salud y políticas públicas, reconociendo la complejidad y la interdependencia de los factores que sustentan la salud mental y emocional a lo largo de la vida^18^. Las intervenciones deben adaptarse a las necesidades específicas de cada población, asegurando que los aspectos emocionales se incorporen eficazmente en los enfoques centrados en la salud y en la CdV.

Un punto central que surge del análisis es que, aunque se puede investigar en diferentes contextos, como los cuidados paliativos^19^, dietas terapéuticas^20^, enfermedades crónicas^21^ o experiencias de minorías culturales^22^ ^-^ ^24^, en todos los casos, el bienestar socioemocional figura como un mediador de la adaptación a la enfermedad y a las adversidades. Esto refuerza la idea de que la salud no puede entenderse únicamente en términos biomédicos, sino que también debe comprenderse como un fenómeno que integra procesos emocionales, relaciones sociales y condiciones estructurales de vida.

Otro aspecto relevante es la dimensión relacional del bienestar socioemocional. Estudios realizados tanto en poblaciones indígenas^22^ ^-^ ^24^ como en contextos clínicos^19^ revelan que la calidad de las conexiones sociales, el apoyo comunitario y la valorización cultural desempeñan un papel central en la preservación del bienestar socioemocional. Esta perspectiva amplía la comprensión del constructo, alejándolo de una lectura individualizada y acercándolo a una visión ecosistémica, en la que la familia, la comunidad y la cultura son factores determinantes.

El debate también pone de relieve la importancia de la equidad en salud como factor determinante del bienestar socioemocional. Estudios^21^ ^,^ ^25^ realizados durante la pandemia de COVID-19 destacan cómo los determinantes sociales, como los ingresos, el acceso a los servicios y el apoyo social, afectan directamente al bienestar socioemocional. Estos hallazgos refuerzan la idea de que las políticas públicas deben considerar que la vulnerabilidad social se traduce no solo en un peor desenlace clínico, sino también en un mayor sufrimiento emocional y social, lo que refuerza la interdependencia entre la desigualdad estructural y la salud mental.

En este contexto, es crucial reconocer que el concepto de bienestar varía significativamente entre individuos y comunidades, moldeado por factores como el contexto sociocultural, las experiencias personales y los valores colectivos. Lejos de ser un concepto estático o universal, el bienestar se configura como una construcción dinámica y situada, cuya comprensión requiere la integración de múltiples perspectivas. Esta variabilidad semántica subraya la importancia de enfoques sensibles a las particularidades de cada grupo^26^.

Cabe resaltar que los estudios analizados revelan que el bienestar socioemocional debe entenderse como un constructo multidimensional y dinámico, atravesado por experiencias de vida, contextos culturales y condiciones sociales^3^. A la vez, estos análisis señalan una importante laguna: la ausencia de instrumentos específicos que midan de forma directa esta dimensión. Esta limitación metodológica no invalida los avances teóricos, pero indica la necesidad de desarrollar medidas más integrales que capten la complejidad del constructo y sirvan como base para las prácticas de cuidado y las políticas públicas encaminadas a promover la salud integral^20^ ^,^ ^24^ ^-^ ^25^.

Una limitación de este análisis de concepto radica en la falta de estandarización terminológica y conceptual en los estudios sobre bienestar socioemocional. La diversidad de definiciones y terminologías utilizadas dificulta la comparación de resultados entre diferentes estudios, lo que compromete la construcción de una base conceptual coherente. Además, el recorte temporal elegido puede haber excluido investigaciones relevantes publicadas fuera del período analizado, lo que limita el alcance de la revisión. Asimismo, el hecho de utilizar únicamente *Google* ^*®*^ Académico como fuente de literatura gris puede haber limitado el acceso a perspectivas complementarias. 

## Conclusión

El bienestar socioemocional es un constructo multidimensional que va más allá de la salud mental individual y abarca las interacciones sociales, emocionales y culturales. Factores como el apoyo social, las relaciones interpersonales, el contexto sociocultural y las condiciones de vida influyen directamente en su manifestación y percepción por parte de los individuos, lo que pone de relieve la necesidad de comprender el concepto de forma integrada y contextualizada.

El análisis de concepto realizado contribuye a consolidar la comprensión del bienestar socioemocional, ofreciendo apoyo para el desarrollo de políticas públicas e intervenciones de cuidado que integren aspectos emocionales, sociales y culturales. Al reconocer la variabilidad del concepto en diferentes contextos y poblaciones, se hace posible promover estrategias más eficaces centradas en la salud integral y en la CdV.

## Data Availability

Todos los datos generados o analizados durante este estudio están incluidos en este artículo publicado
